# Computerised cognitive behaviour therapy (cCBT) as treatment for depression in primary care (REEACT trial): large scale pragmatic randomised controlled trial

**DOI:** 10.1136/bmj.h5627

**Published:** 2015-11-11

**Authors:** Simon Gilbody, Elizabeth Littlewood, Catherine Hewitt, Gwen Brierley, Puvan Tharmanathan, Ricardo Araya, Michael Barkham, Peter Bower, Cindy Cooper, Linda Gask, David Kessler, Helen Lester, Karina Lovell, Glenys Parry, David A Richards, Phil Andersen, Sally Brabyn, Sarah Knowles, Charles Shepherd, Debbie Tallon, David White

**Affiliations:** 1 Department of Health Sciences, University of York, York YO10 5DD, UK; 2 York Trials Unit, Department of Health Sciences, University of York, York YO10 5DD, UK; 3 MRC Epidemiology Unit, University of Cambridge School of Clinical Medicine, Cambridge CB2 0QQ, UK; 4 Department of Population Health, London School of Hygiene and Tropical Medicine, London WC1E 7HT, UK; 5 Centre for Psychological Services Research, Department of Psychology, University of Sheffield, Sheffield S10 2TP, UK; 6 NIHR School for Primary Care Research, Manchester Academic Health Science Centre, University of Manchester, Manchester M13 9NT, UK; 7 Clinical Trials Research Unit, School of Health and Related Research, University of Sheffield, Sheffield S1 4DA, UK; 8 Centre for Academic Primary Care, School of Social and Community Medicine, University of Bristol, Bristol BS8 2BN, UK; 9 Primary Care Clinical Sciences Building, University of Birmingham, Edgbaston, Birmingham B15 2TT, UK; 10 School of Nursing, Midwifery and Social Work, University of Manchester, Manchester M13 9PL, UK; 11 School of Health and Related Research, University of Sheffield, Sheffield S1 4DA, UK; 12 University of Exeter Medical School, University of Exeter, Exeter EX1 2LU, UK; 13 Faculty of Health and Social Care, University of Hull, Hull HU6 7RX, UK

## Abstract

**Study question** How effective is supported computerised cognitive behaviour therapy (cCBT) as an adjunct to usual primary care for adults with depression?

**Methods** This was a pragmatic, multicentre, three arm, parallel randomised controlled trial with simple randomisation. Treatment allocation was not blinded. Participants were adults with symptoms of depression (score ≥10 on nine item patient health questionnaire, PHQ-9) who were randomised to receive a commercially produced cCBT programme (“Beating the Blues”) or a free to use cCBT programme (MoodGYM) in addition to usual GP care. Participants were supported and encouraged to complete the programme via weekly telephone calls. Control participants were offered usual GP care, with no constraints on the range of treatments that could be accessed. The primary outcome was severity of depression assessed with the PHQ-9 at four months. Secondary outcomes included health related quality of life (measured by SF-36) and psychological wellbeing (measured by CORE-OM) at four, 12, and 24 months and depression at 12 and 24 months.

**Study answer and limitations** Participants offered commercial or free to use cCBT experienced no additional improvement in depression compared with usual GP care at four months (odds ratio 1.19 (95% confidence interval 0.75 to 1.88) for Beating the Blues *v* usual GP care; 0.98 (0.62 to 1.56) for MoodGYM *v* usual GP care). There was no evidence of an overall difference between either programme compared with usual GP care (0.99 (0.57 to 1.70) and 0.68 (0.42 to 1.10), respectively) at any time point. Commercially provided cCBT conferred no additional benefit over free to use cCBT or usual GP care at any follow-up point. Uptake and use of cCBT was low, despite regular telephone support. Nearly a quarter of participants (24%) had dropped out by four months. The study did not have enough power to detect small differences so these cannot be ruled out. Findings cannot be generalised to cCBT offered with a much higher level of guidance and support.

**What this study adds** Supported cCBT does not substantially improve depression outcomes compared with usual GP care alone. In this study, neither a commercially available nor free to use computerised CBT intervention was superior to usual GP care.

**Funding, competing interests, data sharing **Commissioned and funded by the UK National Institute for Health Research (NIHR) Health Technology Assessment (HTA) programme (project No 06/43/05). The authors have no competing interests. Requests for patient level data will be considered by the REEACT trial management group

**Trial registration** Current Controlled Trials ISRCTN91947481.

## Introduction

Depression is one of the most common reasons for GP consultations, and its associated personal and economic burden is considerable.^1^ While antidepressants remain an important treatment option, many patients and healthcare professionals would like to access psychological therapy as an alternative or adjunct to drug treatment.^2^ ^3^

Cognitive behaviour therapy (CBT) has emerged as a leading evidence supported form of brief psychological therapy for people with depression.^4^ ^5^ Demand for CBT, however, cannot be met from existing therapist resources.^6^ One alternative to therapist delivered CBT is the provision of therapy through a computer.^7^ Several interactive programmes have been developed that enable CBT to be delivered by computer. National Institute of Health and Care Excellence (NICE) guidelines recommend the provision of computerised CBT (cCBT) as an initial lower intensity treatment for depression as part of a “stepped care” approach in primary care,^5^ and forms one of a range of psychological interventions offered in many Improving Access to Psychological Therapy (IAPT) services.^8^ If effective, such programmes have the potential to expand access to psychological therapy in primary care and could represent an efficient non-pharmacological intervention for depression or adjunct to pharmacological treatments.^9^

For those who decide to use (or commission the provision of) cCBT there are several interactive internet based products; some commercially produced and others free to use.^7^ In the first category, commercial products have been marketed to bodies such as the NHS. The alternative free to use products comprise a range of programmes that have been developed by the public sector or by research institutes. These can be accessed at no direct cost to healthcare providers or patients.

Research evidence in support of cCBT (both commercial and free to use) has generally been supportive,^7^ ^9^ ^10^ with claims of effectiveness comparable with that seen in CBT delivered by a therapist.^9^ ^11^ Meta-analysis of the effectiveness of cCBT has shown larger effect sizes where a level of professional support or guidance is offered to accompany the computer mediated treatment programme.^12^ A concern is the degree to which patients find it acceptable to receive psychological therapy through a computer rather than from a trained therapist. Many patients offered cCBT do not access the material or make minimal use of it.^13^ Non-randomised studies have also shown higher dropout rates^14^ than those seen in summaries of developer led trials.^9^ There has been limited qualitative research into the acceptability of cCBT.^14^ Previous systematic reviews have also highlighted the need for studies that recruit participants in primary care settings (rather than academic centres or secondary care) and the need for longer term follow-up beyond one year and use a standardised diagnostic assessment.^12^

A United Kingdom technology appraisal of computerised treatments published in 2006 gave cautious support to the use of a commercially developed cCBT package for depression but also recommended that an independent evaluation of the acceptability and effectiveness of cCBT be undertaken as a matter of priority.^7^ In 2008 the REEACT trial (Randomised Evaluation of the Effectiveness and Acceptability of Computerised Therapy) was commissioned by the UK National Institute of Health Research (NIHR) Health Technology Assessment (HTA) programme as an independent evaluation. In 2009 the earlier technology appraisal was superseded when cCBT, meeting the stated quality criteria, was generically endorsed in NICE clinical guidelines for the initial treatment of depression.

We investigated the effectiveness and acceptability of supported cCBT as an adjunct to usual GP care for depression and the relative effectiveness of free to use and commercially developed packages. We will report elsewhere the results of a concurrent process evaluation using qualitative methods of the acceptability of supported cCBT.

## Methods

The REEACT study was a pragmatic, multicentre, three arm, parallel, randomised controlled trial. Adults presenting with symptoms of depression in primary care were randomised 1:1:1 to receive either usual care from their GP or usual care from their GP plus one of two interventions: a commercially produced cCBT intervention (“Beating the Blues”) or a free to use cCBT intervention (MoodGYM). Each of these products had previously been endorsed in a technology appraisal^7^ and NICE guidelines^5^ and had been shown to be effective in developer led trials.^15^ ^16^ Appendix 1 shows the trial protocol.

### Recruitment of participants and baseline assessment

We evaluated the use of supported cCBT in the broad population of patients in primary care who were eligible and appropriate for this intervention. We set a minimum eligibility criterion based on a widely used measure of depression severity (score ≥10 on the nine item patient health questionnaire, PHQ-9) as this has been well validated against standardised criteria^17^ and is also the measure commonly used to assess depression and to inform treatment decisions in UK primary care.^18^ We recruited adults (aged ≥18) presenting in primary care with new or existing symptoms of depression (ascertained by PHQ-9), who were not in receipt of cCBT or specialist psychological therapy at the time of recruitment. Potential participants were recruited either directly by their GP or by letter of invitation if their clinical records noted that they had depression. We checked participants’ access to the internet at baseline (before randomisation). Participants either had access to the internet at home or through a close friend or relative. Some participants were happy to access the internet in a central location including a local library, local MIND (a UK based mental health charity), and GP practice (although few participants accessed the internet at these locations).

We excluded patients who were known by their GP to be actively suicidal; experiencing psychotic symptoms; depressed in the postnatal period; or had recently been bereaved. Patients with previous treatment experience of CBT were not excluded.

All participants completed a baseline assessment before randomisation with several self report questionnaires. Participants also completed a diagnostic self reported computer based interview (the clinical interview schedule-revised, CIS-R),^19^ which assesses severity and diagnosis of depression, along with other common mental health disorders, according to ICD-10 (international classification of diseases, 10th revision) criteria.^20^

Participants gave written informed consent before taking part in the study. Recruitment for the trial took place between August 2009 and March 2011 in general practices in York, Manchester, Sheffield, Bristol, Hull, and the northeast of England. Patient safety was monitored by systematic monitoring of adverse events and serious adverse events; each was reviewed by a clinical member of the team for relatedness to trial interventions (in line with an extension to the CONSORT statement^21^). Appendix 2 contains the information sheet given to participants.

### Randomisation, concealment, and blinding

Participants were allocated by simple randomisation to one of three groups without any restrictions placed on the sequence (that is, no blocking or stratification was included in the randomisation procedure). At the point of recruitment we used an automated computer data entry system to conceal treatment allocation from the study researchers. This was administered remotely by the York Trials Unit and used a computer generated code. Because of the nature of the intervention, none of the participants, general practices, or clinicians could be blinded to treatment allocation. GPs were informed by letter of the participant’s treatment allocation.

### Follow-up

We collected follow-up data between December 2009 and April 2013. Participants were asked to provide data at four, 12, and 24 months after randomisation with a series of self completed questionnaires. The primary outcome endpoint was the four month follow-up as this represented the period at which we expected to observe the largest effect between groups.^12^ Data were collected at 12 and 24 months to investigate any longer term outcomes that could be attributed to the intervention.

To maximise retention, researchers arranged telephone or face to face interviews to facilitate data collection at four, 12, and 24 month follow-up points. Participants were sent the questionnaire by post if telephone or face to face contact was not possible. Researchers performing the outcome assessments were not blind to treatment allocation, though observer bias was minimised by the use of self report questionnaires. A monetary voucher was offered to study participants in recognition of time spent in completing follow-up and was non-contingent on response in line with evidence to enhance retention.^22^

### Intervention and comparator (usual GP care)

This was a pragmatic trial. We imposed no constraints on usual GP care in the control or intervention groups, and participants were therefore free during the trial to access any treatment usually available in primary care, including the use of antidepressants, counselling, psychological services (including Improving Access to Psychological Therapy services, which were present in most sites during the course of the trial), or secondary care mental health services.

#### Supported cCBT intervention groups

Participants in the intervention groups were each offered supported cCBT in addition to usual GP care. Participants were encouraged to access their allocated cCBT packages in their own home or at that of a friend/relative with a broadband internet connection. To ensure those without computer access were not denied participation in the REEACT trial, we also gave information on the location of free to use internet connected computers (though few participants used this mode of access).

The cCBT packages were supported by weekly telephone calls to exceed or replicate (by telephone) a level of support offered in earlier developer led trials^16^ ^15^ and in view of the evidence that professionally supported treatment was more likely to be effective than unsupported computer self help programmes.^12^ We also offered a level of support that replicated or exceeded the support offered in routine NHS psychological therapy services in primary care to ensure the results of the REEACT study were generalisable to UK NHS services. Trained technicians delivered the telephone support. Participants in the two intervention groups were encouraged by phone to engage with the course of computerised therapy, and technical issues relating to computers and the online programmes were also resolved. With the participants’ consent we recorded these phone calls to supervise the telephone support staff and to ensure fidelity to this model of technical/motivational support. As part of quality assurance, an experienced trial clinician scrutinised tapes to ensure delivery of technical support in line with the treatment protocol.

#### Experimental group 1

Beating the Blues (Ultrasis, www.ulltrasis.com) is an interactive, multimedia, cCBT package comprising a 15 minute introductory video followed by eight therapy sessions lasting about 50 minutes. The programme is entirely online, and there is no interaction with clinicians or individualised feedback on computer sessions. There are homework exercises between the sessions. Developer led trials have shown that Beating the Blues is efficacious in reducing symptoms of depression.^15^

#### Experimental group 2

MoodGYM (ANU, http://moodgym.anu.edu.au) is a free to use web based CBT programme for depression developed and copyrighted at the Australian National University Centre for Mental Health Research. It consists of five interactive modules, which are made available sequentially on a weekly basis, with revision of all aspects of the programme in the sixth week. The programme is entirely online, and there is no interaction with clinicians or individualised feedback on computer sessions. Developer led trials have shown that MoodGYM is efficacious in reducing symptoms of depression.^16^

We were able to check uptake and online use of each computer programme with reference to computer use records and by self report. We also recorded the number and duration of telephone support calls that were offered and used.

### Outcomes

The primary outcome was the PHQ-9 at the four month follow-up. The PHQ-9 is a self report measure that includes the cardinal cognitive and somatic symptoms of depression as defined by the American Psychiatric Association Diagnostic and Statistical Manual, version four (DSM-IV).^23^ Scores can range from zero to 27, with a recommended cut point of ≥10, which indicates the need for treatment and has been validated against standardised diagnoses of clinical depression.^17^ Severity of depression was reported as continuous PHQ-9 scores and as a dichotomous outcome according to the proportion of participants who were improved with PHQ-9 scores <10. The dichotomised score was set as the primary outcome, and sample size was ascertained on the basis of the ability of the trial to detect differences in the proportion of participants who were improved.

Secondary outcomes included the SF-36 as a measure of health related quality of life. The SF-36 scoring algorithm produces a physical component score (PCS) and mental component score (MCS).^24^ ^25^ We also recorded health state utility with the EQ-5D^26^ and general psychological wellbeing with the clinical outcomes in routine evaluation-outcome measure (CORE-OM). This instrument measures a range of domains including wellbeing, psychological symptoms, function, and risk.^27^

### Patient involvement

Patient and members of the public were involved at several stages of the trial, including the design, management, and conduct of the trial. We received input from patients who had lived with depression and common mental health problems in the design of the trial materials and management oversight through membership of the trial steering committee. A user led organisation (Anxiety UK and Self-Help Services) acted as co-applicant (through its chief executive) and collaborator. We carefully assessed the burden of the trial interventions on patients. We intend to disseminate the main results to trial participants and will seek patient and public involvement in the development of an appropriate method of dissemination.

### Sample size

We designed our trial to test whether computerised CBT represents a clinically effective addition to usual GP care and whether the free to use computerised package did not represent a less effective (non-inferior) choice of therapy for patients. It was powered to capture any benefit of computerised CBT over usual GP care alone and to test the non-inferiority of free to use cCBT. We based our sample size calculation on the usual care arm of primary care depression trials, where the proportion of patients responding to usual care was in the region of 0.6.^28^ This proportion is similar to that found in a UK Health Technology Assessment trial of antidepressants in primary care.^29^ We regarded a figure of not more than 0.15 below this proportion as being acceptable, given the additional care options that are available to patients who do not initially respond to cCBT within a stepped care framework. In our original calculation, to detect non-inferiority with the percentage success in both groups as 60% and a non-inferiority margin of 15% with over 80% power and assuming 25% attrition, we required 200 participants in each of the three arms. Recruitment to the trial went better than expected, and the trial could have finished recruitment early, but there were slightly higher levels of attrition so we re-estimated sample sizes. To retain similar levels of power to detect non-inferiority between the free to use and commercial cCBT programmes, while allowing for 35% attrition, we sought 690 participants (230 participants in each of the three arms). The trial was also powered to detect a difference of 15% between the usual GP care arm and either of the two cCBT arms with a conventional power analysis. The assumption of 80% power (5% two sided significance) to show a difference in the proportion participants improved (PHQ-9<10) of 0.6 versus 0.75 (equivalent to an odds ratio of 2 for not depressed or an odds ratio of 0.5 for depressed) at four months required a sample size of 149 in each group or 230 after allowance for 35% attrition (that is, 690 in total).

### Statistical analysis

We compared outcome measures separately between the following groups of participants: Beating the Blues versus usual GP care (superiority); MoodGYM versus usual GP care (superiority); and MoodGYM versus Beating the Blues (non-inferiority).

For each group comparison, we applied similar analyses depending on type of comparison (superiority or non-inferiority) and the inclusion of potentially important covariates. All analyses were performed in Stata 13, following a pre-specified analysis plan approved by the trial steering committee. All analyses were conducted on an intention to treat basis, with all participants included in the groups to which they were randomised, with two sided significance tests at the 5% significance level for superiority comparisons. All baseline data were summarised by treatment group and reported descriptively, with no formal statistical comparisons.

#### Primary analysis

Groups were compared with a logistic regression model of dichotomised PHQ-9 scores with adjustments for sex, age, severity of depression at baseline, duration of depression, and level of anxiety. We obtained odds ratios and corresponding 95% confidence intervals from this model. For the comparison between Beating the Blues and MoodGYM, we constructed 90% two sided confidence intervals to maintain 5% significance levels throughout. With this method, the free to use cCBT programme MoodGYM would not be inferior to the commercial pay to use cCBT programme Beating the Blues at the 5% level if the upper boundary was below the prespecified margin of non-inferiority (15% difference in proportions, which corresponded with 1.44 for the odds ratio).

#### Secondary analyses

We repeated the primary analysis for the 12 and 24 month dichotomised PHQ-9 data using the same methods as outlined above. We also analysed all time points in one model rather than individual analyses at each time point using a repeated measures multilevel logistic regression model. The values at four, 12, and 24 months were the outcome measures, and the baseline PHQ-9 score, age, sex, duration of depression, level of anxiety, treatment group, and time were included as fixed effects; an interaction between treatment and time was also included in the model. Participants were treated as random effects (to allow for clustering of data within each participant). Different covariance patterns were assessed for the repeated measurements within participants: unstructured, independent, exchangeable, and identity. We estimated overall odds ratios and corresponding 95% (or 90% for non-inferiority) confidence intervals as well as individual odds ratios at each time point (four, 12, and 24 months) from these models. The PHQ-9 in its continuous form, CORE-OM, and SF-36 component scores were analysed with a multilevel linear mixed model following a similar procedure to those outlined above for the dichotomised PHQ-9 scores. The model made adjustments for the same covariates. For continuous outcomes we also reported standard effect sizes (Hedges G).

The statistical analysis and reporting of the REEACT trial followed the CONSORT guidelines.^30^

## Results

One hundred GP practices agreed to take part in the study, with participants being recruited from 83 of these practices. Potential participants were either referred or responded to a letter inviting them to participate in the trial between August 2009 and March 2011. Of 1273 people assessed, 691 eligible and consenting participants were successfully randomised (fig 1). At baseline, 242 people were allocated to the MoodGYM intervention arm, 210 to the Beating the Blues intervention arm, and 239 to the usual GP care arm (unequal numbers were expected by chance under conditions of simple randomisation). Follow-up was achieved for 526 (76%) participants at the four month primary outcome, 484 (70%) at 12 months, and 461 (67%) at 24 months.

**Figure fig1:**
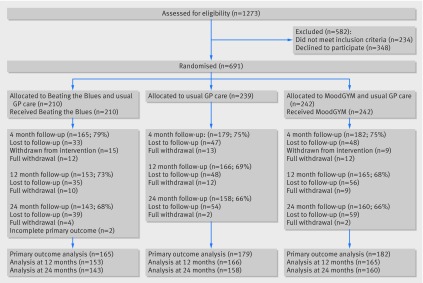
**Fig 1** Selection, randomisation, and flow of participants through trial of computerised cognitive behaviour therapy for depression in primary care

The randomised groups were well balanced and similar on entry to the trial in terms of age, sex, severity of depression, duration of depression, use of antidepressant drugs, and educational attainment (table 1). The median severity of depression across all groups was 17, which broadly equated with a moderate severity of depression.^31^ Over a third of participants had had depression for more than a year. When we applied diagnosis according to the CISR, 81% of participants had a confirmed depressive episode according to ICD criteria, with no difference between groups (82.2% of participants in the Beating the Blues group, 81.1% of participants in the usual GP care group, and 79.9% of participants in the MoodGYM group).

**Table tbl1:** **Table 1** Baseline characteristics of participants in study of computerised cognitive behaviour therapy for depression in primary care. Figures are numbers (percentages) of participants unless stated otherwise

Characteristic at baseline	Beating the Blues (n=210)	Usual GP care (n=239)	MoodGYM (n=242)	Total (n=691)
Mean (SD) age (years)	39.61 (12.34)	40.52 (12.64)	39.43 (12.96)	39.86 (12.65)
Age >65	6 (3)	11 (5)	12 (5)	29 (4)
Women	142 (68)	163 (68)	157 (65)	462 (67)
Depression severity (PHQ-9):				
Mean (SD)	16.78 (4.21)	16.32 (4.52)	16.87 (3.99)	16.65 (4.25)
Median (range)	17 (10-27)	16 (10-27)	17 (10-26)	16. 17 (10-27)
Previous episodes of depression:				
Yes	144 (69)	178 (75)	169 (70)	491 (71)
No	65 (31)	60 (25)	72 (30)	197 (29)
Don’t know	1 (0)	0 (0)	1 (1)	2 (0)
No response	0 (0)	1 (0)	0 (0)	1 (0)
No of episodes of depression:				
No of participants in group	144	178	169	491
1	42 (29)	49 (28)	44 (26)	135 (27)
2	34 (24)	49 (28)	31 (18)	114 (23)
3	12 (8)	21 (12)	23 (14)	56 (11)
4	10 (7)	7 (4)	14 (8)	31 (6)
≥5	22 (15)	24 (13)	22 (13)	68 (14)
Chronically depressed	19 (13)	21 (12)	30 (18)	70 (14)
Don’t know	4 (3)	7 (4)	5 (3)	16 (3)
No response	1 (1)	0 (0)	0 (0)	1 (0)
Previously prescribed antidepressants:				
No of participants in group	144	178	169	491
Yes	129 (90)	152 (85)	149 (88)	430 (88)
No	14 (10)	26 (15)	20 (12)	60 (12)
Don’t know	1 (1)	0 (0)	0 (0)	1 (0)
Seen anyone other than GP for depression:				
No in group	104	129	132	365
Psychiatrist	25 (24)	37 (28)	31 (23)	93 (25)
Psychologist	21 (20)	21 (16)	35 (26)	77 (21)
Counsellor	79 (76)	100 (76)	95 (70)	273 (75)
Community psychiatric nurse	15 (14)	26 (20)	19 (14)	60 (16)
Social worker	2 (2)	3 (2)	1 (1)	6 (2)
CAB	2 (2)	1 (1)	2 (1)	5 (1)
Other statutory/voluntary agency	11 (11)	18 (14)	9 (7)	9 (7)
Don’t know	1 (1)	1 (1)	2 (1)	3 (1)
Duration of depression:				
0 (no problem recorded)	9 (4)	13 (5)	14 (6)	36 (5)
1 (present for <2 weeks)	3 (1)	0 (0)	2 (1)	5 (1)
2 (present for 2 weeks-6 months)	67 (32)	96 (40)	79 (33)	242 (35)
3 (present for 6 months-1 year)	49 (24)	46 (19)	43 (18)	138 (20)
4 (present for 1-2 years)	24 (12)	30 (13)	37 (15)	91 (13)
5 (present >2 years)	56 (27)	53 (22)	64 (27)	173 (25)
Duration of anxiety:				
0 (no problem recorded)	31 (15)	33 (14)	33 (14)	97 (14)
1 (present for <2 weeks)	6 (3)	5 (2)	6 (3)	17 (2)
2 (present for 2 weeks-6 months)	57 (27)	79 (33)	63 (26)	199 (29)
3 (present for 6 months-1 year)	32 (15)	41 (17)	34 (14)	107 (16)
4 (present for 1-2 years)	24 (12)	21 (9)	32 (13)	77 (11)
5 (present >2 years)	58 (28)	59 (25)	71 (30)	188 (27)

### Primary analysis

At four months, 83 of 165 (50%) participants in the Beating the Blues group, 78 of 179 (44%) participants in the usual GP care group, and 89 of 182 (49%) participants in the MoodGYM group remained depressed by the criterion of score ≥10 on the PHQ-9 (table 2). Results from the logistic regression (table 3) showed that there was no statistical evidence of a difference between Beating the Blues and usual GP care at four months (odds ratio 1.19, 95% confidence interval 0.75 to 1.88) or between MoodGYM and usual GP care at four months (0.98, 0.62 to 1.56; table 3). For the non-inferiority comparison between MoodGYM and Beating the Blues, the odds ratio at four months was 0.91 (0.62 to 1.34; P=0.69). The upper 90% confidence limit for the odds ratio was 1.34, thus satisfying statistical criteria for non-inferiority of MoodGYM compared with Beating the Blues. Results of the sensitivity analyses were consistent when we adjusted for centre and when we assessed the impact of drop outs under best (missing=not depressed) and worst case (missing=depressed) scenarios (table 3).

**Table 2 tbl2:** PHQ-9 summary (PHQ-9 ≥10) by duration of follow-up and treatment group. Figures are numbers (percentage) of participants

	Beating the Blues	Usual GP care	MoodGYM
Baseline	210 (100)	239 (100)	242 (100)
4 months ≥10	83/165 (50)	78/179 (44)	89/182 (49)
12 months	54/153 (35)	66/166 (40)	50/165 (30)
24 months	60/143 (42)	61/158 (39)	55/160 (34)

**Table 3 tbl3:** Results from logistic regression and mixed models. Odds ratios are odds of being depressed

Comparison	Odds ratio (95% CI); P value		
	Beating the Blues *v* usual GP care	MoodGYM *v* usual GP care	MoodGYM *v* Beating the Blues
Logistic regression result*			
4 months	1.19 (0.75 to 1.88); 0.46	0.98 (0.62 to 1.56); 0.95	0.91 (0.62† to 1.34†); 0.69
12 months	0.77 (0.47 to 1.26); 0.29	0.56 (0.34 to 0.93); 0.02	0.77 (0.50† to 1.18†); 0.31
24 months	1.00 (0.60 to 1.68); 0.99	0.68 (0.41 to 1.15); 0.15	0.72 (0.47† to 1.11†); 0.21
**Sensitivity analyses at 4 months**			
Adjusted for centre	1.19 (0.83 to 1.71); 0.35	0.98 (0.67 to 1.45); 0.94	0.91 (0.64† to 1.30†); 0.67
Best case scenario (missing=not depressed)	1.34 (0.90 to 2.01); 0.15	1.12 (0.75 to 1.68); 0.58	0.90 (0.65† to 1.27†); 0.62
Worst case scenario (missing=depressed)	1.05 (0.70 to 1.57); 0.83	1.00 (0.68 to 1.49); 0.99	1.03 (0.73† to 1.44†); 0.89
**Mixed model‡**			
4 months	1.27 (0.70 to 2.28); 0.43	1.13 (0.61 to 2.10); 0.70	0.94 (0.57† to 1.55†); 0.84
12 months	0.66 (0.32 to 1.34); 0.24	0.44 (0.22 to 0.88); 0.02	0.73 (0.40† to 1.32†); 0.39
24 months	1.16 (0.44 to 3.05); 0.77	0.62 (0.30 to 1.29); 0.20	0.60 (0.29† to 1.26†); 0.26

*Adjusted for sex, age, baseline depression severity, depression duration and level of anxiety.

†90% confidence interval for non-inferiority comparison.

‡Adjusted for sex, age, baseline depression severity, depression duration, level of anxiety, month, treatment and an interaction between month and treatment as fixed effects.

### Secondary analyses

Results from the PHQ-9 logistic regression highlighted that there was no statistical evidence of a difference between Beating the Blues and usual GP care across any of the time points (odds ratios 0.77 (95% confidence interval 0.47 to 1.26) at 12 months and 1.00 (0.60 to 1.68) at 24 months; table 3 and fig 2). There was some statistical evidence of a difference in favour of MoodGYM versus usual GP care at 12 months (0.56, 0.34 to 0.93), but this was no longer evident at 24 months (0.68, 0.41 to 1.15; table 3 and fig 2). For the non-inferiority comparison, the upper 90% confidence limit for the odds ratio at 12 and 24 months was below the prespecified margin, thus satisfying statistical criteria for non-inferiority (0.77 (0.50 to 1.18) at 12 months and 0.72 (0.47 to 1.11) at 24 months). The results from the multilevel logistic regression model were similar to those from the logistic regression models at each individual time point. There was no evidence of an overall difference between either programme compared with usual GP care (0.99 (0.57 to 1.70) for Beating the Blues *v* usual GP care; and 0.68 (0.42 to 1.10) for MoodGYM *v* usual GP care). The results for the non-inferiority comparison between MoodGYM and Beating the Blues at four months, however, did change: the upper 90% confidence limit for the odds ratio was above the prespecified margin, thus no longer satisfying statistical criteria for non-inferiority with Beating the Blues inferior to MoodGYM (0.94, 0.57 to 1.55).

**Figure fig2:**
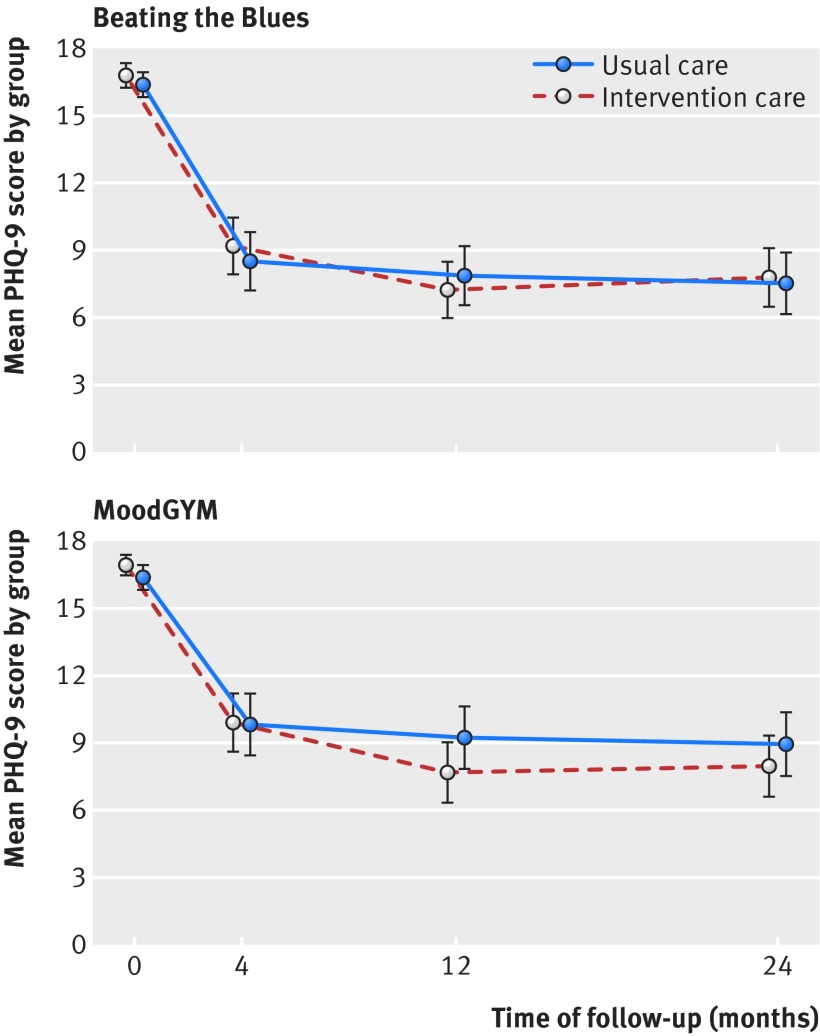
**Fig 2** Depression status measured with PHQ-9 across all follow-up points. Means are all predicted means and 95% confidence intervals estimated from mixed model with sex, age, baseline PHQ-9 score, duration of depression, level of anxiety, month, treatment, and interaction between month and treatment as fixed effects

There was no statistical evidence of a difference on the overall (including all time points) mean depression scores, physical and mental component scores on the quality of life scale (SF-36), and CORE-OM scores for Beating the Blues compared with usual GP care alone (table 4-7 [Table tbl5] [Table tbl6] [Table tbl7]). The standard effect size (Hedges G) for depression scores across all time points for Beating the Blues versus usual GP care alone showed no evidence of a difference (−0.02, 95% confidence interval −0.22 to 0.19; table 4). There was no evidence of a difference on the overall (including all time points) mean depression scores (table 4) and physical component scores on the SF-36 (table 5) for MoodGYM compared with usual GP care alone. The standard effect size (Hedges G) across all time points for MoodGYM versus usual GP care alone was small and not significant (0.09, −0.11 to 0.28; table 4). There was, however, evidence of a difference in favour of MoodGYM for depression scores at 12 months (table 4). There was also evidence of a difference in favour of MoodGYM on the overall (including all time points) mean CORE-OM (table 7) scores and mental component scores on the SF-36 (table 5) compared with usual GP care alone; with evidence of a difference in CORE-OM scores at 12 months and mental component scores on the SF-36 at 12 and 24 months.

**Table 4 tbl4:** Linear mixed model for secondary analyses, PHQ-9 continuous

	Mean (95% CI)*		Difference (95% CI), P value	Effect size (95% CI)
	Intervention	Usual GP care		
**Overall**				
Beating the Blues *v* usual GP care	8.06 (6.92 to 9.20)	7.93 (6.74 to 9.13)	0.12 (−0.87 to 1.11), 0.81	−0.02 (−0.22 to 0.19)
MoodGYM *v* usual GP care	8.49 (7.26 to 9.72)	9.30 (8.00 to 10.60)	−0.81 (−1.75 to 0.13), 0.09	0.09 (−0.11 to 0.28)
**4 months**				
Beating the Blues *v* usual GP care	9.18 (7.94 to 10.42)	8.46 (7.18 to 9.73)	0.72 (−0.46 to 1.90), 0.23	−0.08 (−0.28 to 0.12)
MoodGYM *v* usual GP care	9.86 (8.55 to 11.18)	9.80 (8.42 to 11.18)	0.06 (−1.09 to 1.22), 0.91	−0.01 (−0.20 to 0.19)
**12 months**				
Beating the Blues *v* usual GP care	7.20 (5.94 to 8.46)	7.83 (6.53 to 9.13)	−0.63 (−1.87 to 0.62), 0.33	0.07 (−0.13 to 0.27)
MoodGYM *v* usual GP care	7.63 (6.31 to 8.95)	9.22 (7.83 to 10.60)	−1.59 (−2.75 to -0.42), 0.008	0.16 (−0.03 to 0.36)
**24 months**				
Beating the Blues *v* usual GP care	7.79 (6.47 to 9.12)	7.52 (6.16 to 8.88)	0.28 (−1.10 to 1.65), 0.69	−0.03 (−0.23 to 0.17)
MoodGYM *v* usual GP care	7.97 (6.61 to 9.33)	8.88 (7.45 to 10.30)	−0.91 (−2.16 to 0.35), 0.16	0.09 (−0.11 to 0.29)

*Predicted means (95% CI) from mixed model with sex, age, baseline depression severity, depression duration, level of anxiety, month, treatment, and interaction between month and treatment as fixed effects.

**Table 5 tbl5:** Linear mixed model for secondary analyses, mental component score (MCS)

	Mean (95% CI)*		Difference (95% CI), P value	Effect size (95% CI)
	Intervention	Usual GP care		
**Overall**				
Beating the Blues *v* usual GP care	38.52 (35.77 to 41.27)	37.50 (34.62 to 40.38)	1.02 (−1.34 to 3.37), 0.40	0.05 (−0.16 to 0.26)
MoodGYM *v* usual GP care	38.98 (36.03 to 41.92)	35.99 (32.88 to 39.11)	2.98 (0.73 to 5.24), 0.009	0.14 (−0.06 to 0.34)
**4 months**				
Beating the Blues *v* usual GP care	35.23 (32.28 to 38.17)	36.33 (33.29 to 39.37)	−1.10 (−3.85 to 1.64), 0.43	−0.05 (−0.26 to 0.15)
MoodGYM *v* usual GP care	35.07 (31.90 to 38.23)	34.87 (31.54 to 38.20)	0.20 (−2.60 to 2.99), 0.89	0.01 (−0.19 to 0.21)
**12 months**				
Beating the Blues *v* usual GP care	41.58 (38.55 to 44.60)	38.25 (35.11 to 41.39)	3.32 (0.37 to 6.28), 0.03	0.16 (−0.05 to 0.37)
MoodGYM *v* usual GP care	40.91 (37.77 to 44.05)	36.66 (33.33 to 40.00)	4.25 (1.47 to 7.02), 0.003	0.19 (−0.02 to 0.39)
**24 months**				
Beating the Blues *v* usual GP care	38.76 (35.52 to 41.99)	37.92 (34.62 to 41.22)	0.84 (−2.49 to 4.17), 0.62	0.04 (−0.17 to 0.25)
MoodGYM *v* usual GP care	40.95 (37.68 to 44.23)	36.44 (33.03 to 39.86)	4.51 (1.51 to 7.51), 0.003	0.19 (−0.01 to 0.39)

*Predicted means (95% CI) from mixed model with sex, age, baseline MCS, depression duration, level of anxiety, month, treatment, and interaction between month and treatment as fixed effects.

**Table 6 tbl6:** Linear mixed model for secondary analyses, physical component score (PCS)

	Mean (95% CI)*		Difference (95% CI), P value	Effect size (95% CI)
	Intervention	Usual GP care		
**Overall**				
Beating the Blues *v* usual GP care	48.66 (47.05 to 50.28)	49.55 (47.86 to 51.23)	−0.89 (−2.29 to 0.52), 0.22	−0.08 (−0.29 to 0.13)
MoodGYM *v* usual GP care	48.87 (47.11 to 50.63)	49.91 (48.05 to 51.76)	−1.04 (−2.41 to 0.33), 0.14	−0.08 (−0.28 to 0.12)
**4 months**				
Beating the Blues *v* usual GP care	49.18 (47.44 to 50.92)	49.85 (48.05 to 51.64)	−0.67 (−2.33 to 0.99), 0.43	−0.06 (−0.26 to 0.15)
MoodGYM *v* usual GP care	49.86 (47.97 to 51.75)	50.21 (48.23 to 52.19)	−0.35 (−2.04 to 1.33), 0.68	−0.03 (−0.23 to 0.18)
**12 months**				
Beating the Blues *v* usual GP care	48.61 (46.80 to 50.43)	49.73 (47.86 to 51.61)	−1.12 (−2.95 to 0.71), 0.23	−0.09 (−0.30 to 0.12)
MoodGYM *v* usual GP care	48.85 (46.96 to 50.73)	50.06 (48.07 to 52.05)	−1.21 (−2.91 to 0.49), 0.16	−0.09 (−0.29 to 0.11)
**24 months**				
Beating the Blues *v* usual GP care	48.19 (46.17 to 50.22)	49.06 (47.02 to 51.10)	−0.87 (−3.06 to 1.32), 0.44	−0.06 (−0.27 to 0.15)
MoodGYM *v* usual GP care	47.89 (45.82 to 49.97)	49.45 (47.30 to 51.60)	−1.56 (−3.61 to 0.50), 0.14	−0.11 (−0.31 to 0.10)

*Predicted means (95% CI) from mixed model with sex, age, baseline PCS, depression duration, level of anxiety, month, treatment, and interaction between month and treatment as fixed effects.

**Table 7 tbl7:** Linear mixed model for secondary analyses, CORE-OM

	Mean (95% CI)		Difference (95% CI), P value	Effect size (95% CI)
	Intervention	Usual GP care		
**Overall**				
Beating the Blues *v* usual GP care	11.74 (10.27 to 13.21)	12.18 (10.64 to 13.71)	−0.44 (−1.70 to 0.82), 0.50	0.04 (−0.17 to 0.25)
MoodGYM *v* usual GP care	11.98 (10.47 to 13.50)	13.14 (11.55 to 14.73)	−1.16 (−2.31 to 0.002), 0.05	0.11 (−0.10 to 0.31)
**4 months**				
Beating the Blues *v* usual GP care	12.87 (11.31 to 14.43)	12.75 (11.14 to 14.36)	0.12 (−1.33 to 1.57), 0.87	−0.01 (−0.22 to 0.20)
MoodGYM *v* usual GP care	13.61 (12.01 to 15.20)	13.69 (12.02 to 15.36)	−0.09 (−1.44 to 1.27), 0.90	0.01 (−0.19 to 0.21)
**12 months**				
Beating the Blues *v* usual GP care	10.51 (8.91 to 12.11)	12.00 (10.34 to 13.66)	−1.49 (−3.04 to 0.06), 0.06	0.13 (−0.07 to 0.34)
MoodGYM *v* usual GP care	10.87 (9.25 to 12.48)	12.98 (11.28 to 14.68)	−2.12 (−3.54 to −0.69), 0.004	0.18 (−0.02 to 0.38)
**24 months**				
Beating the Blues *v* usual GP care	11.84 (10.13 to 13.55)	11.78 (10.05 to 13.51)	0.06 (−1.68 to 1.80), 0.95	−0.01 (−0.21 to 0.20)
MoodGYM *v* usual GP care	11.48 (9.80 to 13.16)	12.74 (11.00 to 14.49)	−1.27 (−2.80 to 0.27), 0.11	0.11 (−0.10 to 0.31)

*Predicted means (95% CI) from mixed model with sex, age, baseline CORE-OM, depression duration, level of anxiety, month, treatment, and interaction between month and treatment as fixed effects.

### Participant safety

In total, 302 participants reported 745 non-serious adverse events: 93 participants (264 events) in the Beating the Blues group, 110 participants (241 events) in the usual GP care group, and 99 participants (240 events) in the MoodGYM group. Table 8 summarises adverse events by trial arm. Across all participants, 49 serious adverse events were recorded from 39 participants. Of these, 39 were judged to be unrelated and nine unlikely to be related to the trial intervention. We were unable to make a judgment of relatedness to the trial intervention for one serious adverse event reported because of limited information provided. Five of the serious adverse events were referred to the data monitoring and ethics committee for discussion, and two were referred to the research ethics committee. Of the 49 serious adverse events, 40 (82%) involved inpatient admission to hospital, seven were life threatening, and two resulted in participants dying.

**Table 8 tbl8:** Summary of adverse events (non-serious (NSAE) and serious (SAE)). Figures are numbers (percentage) of participants

	Beating the Blues (n=210)	Usual GP care (n=239)	MoodGYM (n=242)	Total (n=691)
**Non-serious adverse events**				
Total	264	241	240	745
No of participants with ≥1 event	93 (44)	110 (46)	99 (41)	302 (44)
No per patient:				
1	27 (29)	49 (45)	36 (36)	112 (37)
2	30 (32)	31 (28)	21 (21)	82 (27)
3	14 (15)	13 (12)	22 (22)	49 (16)
4	6 (7)	7 (6)	12 (12)	25 (8)
5	5 (5)	6 (6)	6 (6)	17 (6)
≥6	11 (12)	4 (4)	2 (2)	17 (6)
**Serious adverse events**				
Total	19	19	11	49
No of participants with ≥1 event	15 (7)	15 (6)	9 (4)	39 (6)
Events per patient:				
1	12 (80)	11 (73)	7 (78)	30 (77)
2	2 (13)	4 (27)	2 (22)	8 (21)
3	1 (7)	0	0	1 (3)
Relation to treatment:				
Unrelated	18 (95)	15 (79)	6 (55)	39 (80)
Unlikely to be related	0	4 (21)	5 (46)	9 (18)
Possibly related	0	0	0	0
Probably related	0	0	0	0
Definitely related	0	0	0	0
Unable to assess*	1 (5)	0	0	1 (2.0)
Referred to DMEC	0	3 (16)	2 (18)	5 (10)
Referred to REC	0	2 (11)	0	2 (4)
Event details:				
Involved inpatient admission†	17 (90)	13 (68)	10 (91)	40 (82)
Life threatening†‡	0	6 (32)	1 (9)	7 (14)
Patient died	2 (11)	0	0	2 (4)
Involved persistent or considerable disability or incapacity‡	0	0	0	0
Resulted in congenital anomaly or birth defect	0	0	0	0

DMEC=data monitoring ethics committee, REC=research ethics committee.

*Researcher was informed that participant had recently been admitted to hospital but when contacted participant did not provide further information and withdrew from study.

†One SAE was classed as both involving inpatient admission and life threatening.

‡One SAE was classed as both life threatening and involving persistent or considerable disability or incapacity.

### Uptake and use of computerised CBT and telephone support

After allocation, 83% (n=175) of Beating the Blues participants and 77% (n=186) of MoodGYM participants accessed the programmes (as ascertained by computer login records). When asked at four months after randomisation, 19% of the usual GP care group reported having used computerised CBT; either on the recommendation of their GP, by self referral, or at the recommendation of a mental health professional.

From computer verified records, we ascertained that the median number of sessions completed for Beating the Blues was two (interquartile range 0-5), and the most commonly used (median) number of sessions was one (out of eight computer sessions). For MoodGYM the median number of sessions completed was one (0-2), and the most commonly used number of sessions was one (out of six computer sessions). Of those participants who started the programmes, 31 participants (18%) completed all eight sessions of Beating the Blues, and 29 participants (16%) completed all six sessions of MoodGYM.

We attempted a median of 13 contacts to participants allocated to Beating the Blues (interquartile range 11-16) and MoodGYM (10-16). A total mean of 6.0 (3-8) technical telephone support calls were made to Beating the Blues participants and 6.8 (4-9) to MoodGYM participants. Of the technical telephone support calls made, the mean number of calls answered by participants was 3.1 (1-5) for Beating the Blues participants and 3.3 (1-5) for MoodGYM participants. The total mean number of minutes of technical support calls delivered to participants was 6.2 (2-8) minutes for Beating the Blues participants and 6.5 minutes (2-9) for MoodGYM participants. The mean number of emails sent was 5.3 (2-8) for Beating the Blues participants and 5.0 (1-8) for MoodGYM participants. Texts were rarely sent (mean of <0.1 texts sent to both Beating the Blues and MoodGYM participants).

### Usual care received

Examination of GP medical records showed that participants received a range of treatments as part of the usual care from their GP (table 9). Participants received a range of antidepressant drugs and access to various mental health services, including referral to Improving Access to Psychological Therapy services, community mental health teams, psychiatrists, psychologists, and counsellors. Such services were used by similar proportions of participants across each of the three groups. In addition, participants visited their GP to a comparable extent across the three groups. We present the results of a full economic evaluation elsewhere.^32^

**Table 9 tbl9:** Primary and secondary care services accessed by trial participants from baseline to 12 month follow-up (data obtained through GP medical records)

Service	Beating the Blues (n=173)			Usual GP care (n=202)			MoodGYM (n=205)	
	Mean (SD)	Used by (%)		Mean (SD)	Used by (%)		Mean (SD)	Used by (%)
**Primary care**								
GP	7.66 (5.48)	96		6.94 (4.82)	96		6.98 (4.62)	98
Nurse	1.60 (2.31)	58		1.86 (2.85)	63		2.03 (3.86)	61
Out of hours	0.09 (0.33)	8		0.07 (0.31)	6		0.06 (0.26)	5
**Medication**								
Depression related	NA	82		NA	84		NA	77
**Secondary care**								
IAPT	0.25 (1.23)	5		0.41 (1.25)	13		0.33 (1.46)	7
CMHT	0.20 (1.58)	5		0.14 (0.70)	6		0.08 (0.41)	4
Counsellor	0.28 (1.65)	5		0.04 (0.29)	2		0.13 (0.86)	3
Psychiatric	0.17 (1.16)	5		0.13 (0.82)	3		0.11 (0.88)	2
Psychological	0.46 (2.95)	4		0.37 (2.26)	4		0.01 (0.21)	1

NA=not applicable; IAPT=Improving Access to Psychological Therapy (NHS service offering psychological interventions to individuals with depression and anxiety disorders); CMHT=community mental health teams.

## Discussion

### Principal findings

The REEACT trial showed that for the primary outcome of severity of depression at four months there was no significant benefit for supported cCBT in addition to usual GP care. Confidence intervals were wide for these estimates, and there was 24% drop out at four months. The estimates and 95% confidence intervals at our primary time point of interest were all larger than our original sample size estimate (odds ratio 0.5). This negative finding was true for both a free to use package (MoodGYM) and commercially produced cCBT (Beating the Blues). We found a significant benefit for cCBT in a secondary analysis for mental health quality of life and generic psychological wellbeing at 12 months after treatment. These secondary outcomes favoured the free to use package, but no consistent effect was seen for commercially produced cCBT at all but one time point for one outcome (mental health quality of life at 12 months). It is questionable whether this would be seen as clinically important and was not observed for the primary outcome of four months nor was it evident at longer term follow-up.

To our knowledge the REEACT trial is the largest pragmatic independent evaluation of the effectiveness of supported cCBT in primary care. The trial is novel in that we examined a question that is important to commissioners of services by comparing packages that were free to use versus commercially available products. Both these products were recommended in depression guidelines issued by NICE at the time we designed the REEACT trial and were still endorsed by NICE at the time of publication.^5^ Our main finding is therefore that while cCBT has been shown to be efficacious in developer led trials, it was not effective in usual NHS care settings. The main reason for this was low adherence and engagement with treatment, rather than lack of efficacy.

The REEACT trial included an extended follow-up to 24 months. Neither of the supported cCBT interventions showed any benefit over usual GP care when all time points were taken into account in a repeated measures analysis, although there was a small but significant benefit for MoodGYM at 12 months’ follow-up. By 24 months there was no statistical evidence of a difference between intervention and control. The small benefit for MoodGYM at 12 months is therefore difficult to interpret and is not in keeping with other research in this specialty, where benefit is usually observed only in the short term.^33^

### Comparison with other studies

The results of the REEACT trial are different from those from developer led trials, which have recently been summarised in systematic reviews^9^ ^10^ and technology appraisals.^7^ Aside from the independence of the REEACT evaluation from those who have developed cCBT, there are several differences in design that are worthy of note. The first is that the REEACT trial was purposely conducted entirely within primary care, which is the setting in which most people with depression receive treatment.^34^ This contrasts with several trials that have recruited their target population through the internet or in specialist (secondary care) referral centres or in centres that have developed specialist clinics where participants are directly supervised while they use a computer package. We would argue that this enhances the degree to which the REEACT results can be applied to primary care and to mental health services led by primary care.

The second difference is the level of support that was offered. In the REEACT trial participants were proactively offered a high level of technical support and weekly encouragement to use the computer packages, but we purposely did not augment structured psychological therapy over the telephone using trained psychological therapists. Telephone support in the REEACT trial did not involve detailed explanations of cognitive behavioural therapy and did not involve detailed review of homework or tasks between sessions. The cCBT was therefore a form of supported self help but was not one that was guided by a clinician. Systematic reviews have shown that unsupported self help treatment (including unsupported computer delivered self help) has minimal effects and a relatively small effect size.^12^ ^35^ In contrast, efficacy trials have shown that more intensively supported treatments generally have moderate effect sizes claimed to be comparable with face to face therapy.^11^ The level of support in the REEACT trial is one that is at least as intensive as that offered in many NHS care settings and is in line with (or in the case of MoodGYM exceeds) the level of support that is generally offered.^16^ The REEACT trial therefore represents an evaluation of a supported intervention that replicates the use of cCBT in routine primary care settings. The chosen level of support, however, was less intense than other efficacy trials in which computer use has, for example, been supervised on a 1:1 basis by therapists or in which a healthcare professional has been physically present to ensure the user interacts successfully with the computer (see, for example, Proudfoot and colleagues^15^). More intensively supported cCBT might have been more likely to have resulted in a positive outcome but would have diluted the pragmatic evaluation of the effectiveness of cCBT as it is offered in primary care. Because there was weekly proactive telephone support, we expected that this effectiveness trial would show some benefit comparable with that observed in developer led guided self help trials.^12^ Future research could explore whether the use of telephone delivered clinician support results in a higher level of engagement and a more effective form of computerised treatment. An alternative mode of support is by the use of online therapists, which has shown positive results in primary care.^36^

Our other significant finding was that, despite the provision of a high level of telephone support, there was relatively low uptake of computerised CBT. This finding is in line with other lower intensity forms of intervention for depression offered in primary care, where dropout and failure to engage with therapy are common.^37^ It contrasts, however, with other developer led efficacy trials in which good levels of engagement and uptake have been reported.^37^

Again we note that REEACT was a pragmatic study, in which a feasible and representative primary care intervention was replicated within the context of a randomised controlled trial. The trial was a real world evaluation of supported computer delivered CBT. The reasons for poor engagement and barriers to the use of this technology in routine care have hitherto not been explored within the context of trials, and a companion paper reports in depth the reasons for poor engagement.^38^ We have found that participants offered cCBT were generally unwilling to engage with computer programmes and highlighted the difficulty in repeatedly logging on to computer systems when they are clinically depressed. Participants said they wanted a greater level of clinical support as an adjunct to therapy, and, in the absence of this support, they commonly disengaged with the computer programmes.

The REEACT study was also pragmatic in reflecting the degree to which GPs might offer cCBT for people with a range of severities of depression. Though we set a minimum entry criterion (PHQ-9 ≥10) we noted that the mean score was 17, which is indicative of moderately severe depression. This is a slightly higher level of severity than is recommended in NICE guidance, and an important finding is that GPs seem ready to offer cCBT as a treatment at a range of severities. This was often in combination with treatment with antidepressants. Future research could evaluate cCBT within a more constrained range of severity of depression; though we noted that use was low for people with all severities of depression.

### Limitations of REEACT trial

There were limitations to this study. Firstly, participants were selected with a definition of depression based on a cut point drawn from a depression severity scale.^17^ We did not use a diagnosis of depression based on a structured diagnostic interview schedule as the primary inclusion criterion. REEACT, however, was a pragmatic randomised controlled trial, and we deliberately adopted a criterion drawn from an instrument that is widely used in UK primary care to guide treatment.^18^ We did, however, concurrently use a diagnostic interview schedule, and 81% or our participants met the ICD criteria for depressive episode. Secondly, REEACT was powered at 80% to detect a modest effect size that is comparable with other low intensity forms of psychological therapy observed in primary care settings.^37^ We did not have sufficient statistical power to detect much smaller effect sizes that have been observed in entirely unsupported cCBT.^35^ Nevertheless, REEACT was a supported form of cCBT, and our trial exceeded 80% statistical power to detect the range of effect sizes that have been reported in systematic reviews of previous trials of cCBT.^12^

We also note that there was potential crossover, with some reported access of computer based treatment or support by 19% participants in the usual care arm of the REEACT trial. Unfortunately we do no not know what form of support was accessed, and this could include a range of computer materials (including cCBT programmes). From the intervention arms, we were able to access the computer records to ascertain actual use of the technology, and this was low even for people who self reported accessing cCBT. As a pragmatic study, usual care was not constrained in REEACT, and this was a strength of the design. It is unlikely that crossover and dilution of effect is a sufficient explanation of the negative findings in REEACT, and the more likely explanation is lack of uptake of the intervention when this is offered, even with telephone support.

### Conclusions and policy implications

Computerised CBT forms a core component of psychological therapy services in primary care in the UK and other health systems. The overall conclusion is that supported computerised cognitive behaviour therapy confers modest or no benefit over usual GP care and suggests that the routine promotion and commissioning of cCBT be reconsidered in light of our findings. Commercially developed computerised cognitive behaviour therapy products confer little or no benefit over free to use products. This is an important finding for those who commission services and purchase commercial products on behalf of publicly funded health services. The routine use and purchase of computerised therapy is likely to be an ineffective form of low intensity treatment for depression and an inefficient use of finite healthcare resources. There is a range of treatments for depression that might be considered instead of cCBT. These include telephone guided self help, bibliotherapy, low intensity psychological workers supporting self help technologies, and therapist delivered cognitive behaviour therapy.^5^ There is a need to evaluate the clinical and cost effectiveness of such approaches. In relation to cCBT there is, for example, an ongoing independent trial (REEACT 2) of the effectiveness of guided computer therapy versus the effectiveness of guided bibliotherapy for depression.

**What is already known on this topic**

There is an increasing interest in the delivery of cognitive behaviour therapy (CBT) through computers (cCBT), which is a potentially effective and efficient mode of delivery for the large numbers of people with depression in primary care
cCBT is endorsed in evidence supported NICE guidelines and forms a component of Improving Access to Psychological Therapy services, but research has generally been conducted in specialist centres and by researchers who have also developed the programmes

**What this paper adds**

This study was a large independent evaluation of the effectiveness of commercial and free to use cCBT in UK primary care
Despite the provision of telephone support to use the cCBT programmes, there was limited uptake by people with clinical depression
Commercially developed and free to use cCBT programmes conferred little or no clinical benefit when offered in addition to usual primary care for depression

## Notes

We thank the REEACT participants for their valuable contribution to this study; Rachel Richardson, who established and assisted with the management of the trial; the research teams at each of our research sites; the GPs, research, and support staff at all GP practices; the Primary Care Research Network and the Mental Health Research Network; and members of the trial steering committee and data monitoring and ethics committee. We also thank David Torgerson for his valuable contribution to the design of the study and insightful advice throughout the trial, and Stephen Palmer, Ana Duarte, and Simon Walker for designing and conducting the economic evaluation (to be reported elsewhere); our patient and public contributors for their valuable input throughout the trial; and the developers of MoodGYM and Ultrasis for providing us with participant log-ins and use data.

The REEACT trial is dedicated to the memory of Professor Helen Lester (1961-2013) who contributed time and wisdom at every stage of the REEACT trial.

Contributors: SG was the chief investigator, initiated the collaborative project, designed the trial, provided management oversight of the whole trial, chaired the trial management group (TMG), drafted and revised the paper, and is guarantor. EL was the trial manager, monitored data collection for the whole trial, and drafted and revised the paper. CH was the trial statistician, wrote the statistical analysis plan, conducted all statistical analyses and drafted and revised the paper. GB was the trial manager, monitored data collection for the whole trial, and provided technical telephone support to trial participants. PT was the trial manager and monitored data collection for the whole trial. RA, MB, PB, CC, LG, DK, HL, KL, GP, and DAR contributed to the trial design, assisted with the conduct of the trial at their respective sites and were members of the TMG. PA provided technical telephone support to trial participants. SB, SK, CS, DT and DW were site trial coordinators and monitored data collection in their respective sites. All authors contributed to redrafts of the report. All authors had full access to all of the study data and take responsibility for the integrity of the data and the accuracy of the data.

Ethical approval: The Leeds (East) research ethics committee approved the trial on 10 July 2008 (08/H1306/77). All participants provided written informed consent to participate in the trial. The full trial protocol is provided in appendix 1 and can also be accessed at www.nets.nihr.ac.uk/projects/hta/064305.

Transparency: The lead author affirms that this manuscript is an honest, accurate, and transparent account of the study being reported; that no important aspects of the study have been omitted; and that any discrepancies from the study as planned (and if relevant registered) have been explained.

Data sharing: Reasonable requests for patient level data should be made to the corresponding author and will be considered by the REEACT trial management group. Consent for data sharing was not obtained but the presented data are anonymised and risk of identification is low.

## Notes

Cite this as: *BMJ* 2015;351:h5627
