# Supplementary material for: Computerised cognitive behaviour therapy (cCBT) as treatment for depression in primary care (REEACT trial): large scale pragmatic randomised controlled trial
Source: BMJ. 2015 Nov 11;351:h5627. doi: 10.1136/bmj.h5627 (PMC4641883; doi:10.1136/bmj.h5627)
Supplement: Supplementary file 1 [file gils026474.ww1_default.pdf]

Trial Protocol

The Randomised Evaluation of the Effectiveness and Acceptability of Computerised Therapy (REEACT) Trial

Protocol Number: 250101
ISRCTN91947481
Sponsor: University of York

Signature Page:

Chief Investigator:

Professor Simon Gilbody

Alcuin C Block
Department of Health Sciences
University of York
York
YO10 5DD

Tel: 01904 321370
Email: simon.gilbody@york.ac.uk

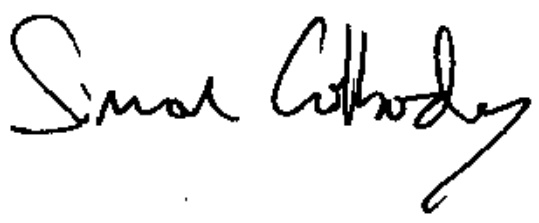

10th December 2012

Signature Date

Co-Investigators (Site Principal Investigators in Bold)

Bristol

Professor Ricardo Araya
Professor of Psychiatry
Academic Unit of Psychiatry
University of Bristol
Cotham House
Cotham Hill
Bristol
BS6 6JL
Tel: 0117 331 4028
Email: r.araya@bristol.ac.uk

Dr David Kessler
Senior Research Fellow in Primary Care
Academic Unit of Primary Health Care
University of Bristol
Cotham House
Cotham Hill
Bristol
BS6 6JL
Tel: 0117 331 4031
Email: david.kessler@bristol.ac.uk

Manchester

Dr Peter Bower
Reader
National Primary Care Research and Development Centre
University of Manchester
Manchester
M13 9PL
Tel: 0161 275 7638
Email: peter.bower@man.ac.uk

Professor Linda Gask
Professor of Primary Care Mental Health
National Primary Care Research and Development Centre
University of Manchester
Manchester
M13 9PL
Tel: 0161 275 7638
Email: linda.gask@man.ac.uk

Professor Helen Lester
Professor of Primary Care
Primary Care Clinical Sciences Building
University of Birmingham
Edgbaston
Birmingham
B15 2TT
Tel: 0121 414 2684
Email: h.e.lester@bham.ac.uk

Professor Karina Lovell
Professor of Mental Health
School of Nursing, Midwifery and Social Work
University of Manchester
Manchester
M13 9PL
Tel: 0161 306 7853
Email: karina.lovell@man.ac.uk

Nicola Lidbetter
Manager, National Phobics' Society
Zion CRC
339 Stretford Road
Hulme
Manchester
M15 4ZY
Tel: 08444 775774
Email:n.lidbetter@ntlworld.com

Sheffield

Professor Michael Barkham
Professor of Clinical Psychology
Director, Centre for Psychological Services Research
Clinical Psychology Unit/ScHARR
Department of Psychology
Western Bank
University of Sheffield
Sheffield S10 2TP
Tel: +44(0)114 222 6527
Email: m.barkham@sheffield.ac.uk

Dr Cindy Cooper
Senior Research Fellow: Clinical Trial
ScHARR
University of Sheffield
Regent Court
30 Regent Street
Sheffield
S1 4DA
Tel: 0114 222 0743
Email: c.l.cooper@sheffield.ac.uk

Professor Glenys Parry
Professor of Psychological Therapies
ScHARR
University of Sheffield
Regent Court
30 Regent Street
Sheffield
S1 4DA
Tel: 0114 222 5454
Email: g.parry@sheffield.ac.uk

York

Professor Simon Gilbody
Department of Health Sciences
Alcuin C Block
University of York
York
YO10 5DD
Tel: 01904 321370
Email: simon.gilbody@york.ac.uk

Mr Steven Palmer
Senior Research Fellow
Centre for Health Economics
University of York
York
YO10 5DD
Tel: 01904 321401
Email: stephen.palmer@york.ac.uk

Professor David Torgerson
Director, York Trials Unit
ARRC
Department of Health Sciences
University of York
York
YO10 5DD
Tel: 01904 321340
Email: david.torgerson@york.ac.uk

Professor Mark Sculpher
Professor of Health Economics
Centre for Health Economics
University of York
York
YO10 5DD
Tel: 01904 321401
Email: mjs23@york.ac.uk

Mrs Rachel Richardson
Research Fellow
Department of Health Sciences
University of York
York
YO10 5DD
Tel: 01904 321863
Email: rachel.richardson@york.ac.uk

Exeter

Professor David Richards
Professor of Mental Health Services
Research
School of Psychology
University of Exeter
Room 118, Washington Singer
Building
Perry Road
Exeter
EX4 4QG
Tel: 01392 264615
Email: d.a.richards@exeter.ac.uk

1. Background

Cognitive behaviour therapy (CBT) has emerged as the leading evidence-supported form of brief psychotherapy for people with depression. [1][2]However, it is unfeasible that demand for CBT can be met from existing therapist resources. [3] Primary care doctors therefore have relatively few treatment options other than antidepressant medication and/or referral to specialist psychology services where long waiting lists are likely.

Computerised CBT represents an alternative form of therapy delivery that has the potential to enhance access to psychological care. Existing research into computerised CBT has most recently been summarised by Kaltenthaler *et al* in their 2006 review of clinical and cost effectiveness. [4]With respect to depression, three commercially-produced computerised packages available to the NHS were considered – *Beating the Blues (BtB)*, *Cope* and *Overcoming Depression*. Of these only one, *BtB*, had been evaluated in a randomised controlled trial. [5] However, this research was conducted by those who owned and held intellectual copyright to *BtB*. Amongst internet-based free-to-use packages, only one, *MoodGYM,* has been evaluated in a randomised trial, also conducted by the package developers. [6] The overall conclusion of the HTA review was that *'the efficacy but not effectiveness of Beating the Blues had been established in comparison with treatment as usual'*. [4] However, several caveats applied and specific recommendations for further research were made that are important with respect to the present trial protocol.

1. The cost effectiveness of computerised packages is unclear. More importantly, the cost effectiveness from the perspective of the UK NHS has not been sufficiently established and the longer term cost effectiveness beyond the brief time horizon of existing trials is essentially unknown. This is important since commercial packages (such as *BtB*) will need to be purchased at substantial cost to the NHS. The major burden of costs associated with depression have been highlighted by Layard, [7] and are at a societal level (lost employment and increased welfare costs). The cost effectiveness of computerised CBT from a societal perspective is unknown.
2. Existing trials use highly selected populations who are necessarily comfortable with information technology and willing to be randomised to computerised therapy as a treatment option. The acceptability of the replacement of the therapist with a machine-interface is largely unknown.
3. There are no trials of free-to-use computerised CBT packages versus commercial computerised CBT. Similarly, there are no trials of computerised CBT versus therapist-led CBT. This is important since the effectiveness of free-to-use computerised CBT would need to be comparable to pay-per-use CBT or therapist-led CBT if it were to be a viable alternative within a stepped care pathway. [8]
4. Evaluations of all the commercially available and free to use packages of computerised CBT have been conducted by companies or

researchers responsible for their development. Whilst this does not invalidate the results, it does raise concerns that a truly independent evaluation of the clinical and cost effectiveness of computerised CBT is needed to inform NHS decision making. Kaltenthaler et al make this a core research recommendation, where they state: *'Research needs to be carried out by independent researchers. It should be carried out by those who are not associated with commercial or product gains.'* [4]

The present trial is designed to address these recommendations. Two products (*MoodGYM and BtB*) have demonstrated efficacy in a primary care setting. This study will represent Phase III of the MRC Complex Interventions Framework [9] and will be a definitive evaluation of computerised CBT in a trial that is adequately controlled and has appropriate statistical power. The post trial modelling phase corresponds to Phase IV of the MRC framework. In this phase we plan to answer important questions regarding generalisability and long term cost effectiveness. We will also conduct a qualitative process evaluation of the acceptability of this new technology to users and to the NHS.

2. Research Objectives

This will be a fully randomised trial of usual GP care for depression versus the addition of one of two computerised CBT packages to usual GP care. We will include a concurrent economic and qualitative evaluation to meet the following specific aims:

1. To establish the clinical and cost effectiveness of the addition of computerised CBT to usual GP care over a two year trial follow-up period, and to assemble a primary care depression cohort of trial patients with a follow-up period of up to 10 years.
2. To establish the acceptability (to patients and clinicians) of computerised CBT.
3. To establish the differential clinical and cost effectiveness of a free-to-use computerised package, in comparison to a commercial pay-to-use computerised CBT package over a two year and longer-term time horizon.

3. Methods

3.1 Design

3.1.1 Trial-based clinical and economic evaluation: This will be a fully randomised controlled trial to evaluate the clinical and cost effectiveness of computerised CBT packages when added to usual GP care. Patients who meet our pragmatic inclusion criteria will be individually randomised into one of three treatment groups: (1) usual GP care PLUS a commercial pay-to-use computerised CBT package (*BtB*); (2) usual GP care PLUS a free-to-use computerised CBT package (*MoodGYM*) and (3) usual GP care. All patients

will therefore receive usual GP care, and we anticipate that the majority of patients in the usual care group will receive antidepressants in line with NICE guidance. Our evaluation is therefore a pragmatic trial. The key comparison will be in the additional benefits that might be expected through the early addition of computerised CBT to usual care. There will be a concurrent economic evaluation whereby we will collect patient-level resource and service-use data to determine the comparative cost effectiveness of commercial versus free-to-use CBT – each in comparison with usual GP care.

The issue of patient preference for either computerised therapy or usual care may well be important in determining their relative effectiveness. [4][10] Computerised CBT offers the potential of greater access to therapy, since it is not constrained by finite therapist numbers. However, a key dimension in the clinical effectiveness of computerised CBT will be the acceptability of therapy delivered by computer. Conversely, successful computerised CBT requires a level of time and commitment on the part of the patient, whereas usual GP care (focussed mainly on drug therapy) may be more acceptable for patients (who may not be sufficiently motivated to engage in computerised CBT). The research into the acceptability of and preference for computerised CBT in general is scarce. [10]

Conventional trial designs ignore the issue of patient preference, giving an 'average' treatment effect for all those who are willing to undergo randomisation. The REEACT trial will use a 'fully randomised preference approach'[11] to examine the issue of patient preference and the differential impact of preference on the relative effectiveness of usual care versus computerised CBT. Participants will each be asked about their baseline preference for either usual care plus computerised CBT or usual care prior to randomisation, and this will be further examined within a planned subgroup analysis. This strategy preserves the advantages of randomisation (unconfounded estimates of effect and the ability to draw causal inference from our trial), whilst still studying the impact of patient preference. [11] Patient preference designs are rarely employed in mental health, [12] and an important output of the REEACT trial will be to fully integrate the use of preference designs in a new and innovative area of research.

3.1.2 Concurrent process evaluation of the acceptability and implementation of computerised CBT: There has been little previous qualitative work exploring issues of the acceptability of computerised CBT, particularly from a primary care perspective. A systematic review by Waller and Gilbody of barriers to computerised CBT found only five relevant qualitative papers, all of relatively poor quality. [10] The review highlighted practical issues such as the substantial numbers of potential participants lost prior to trials commencing, the generally positive views of GPs about the introduction of computerised CBT and problems created by variable patient computer literacy. Drop out rates of up to 25% were also an issue in a previous evaluation of computerised CBT. [6] The impact of computerised therapies with or without internet support will, however, become an increasingly important issue both within and beyond the arena of mental

health[10] and therefore this process evaluation of the REEACT trial will provide important generalisable information for the wider health community.

We will use May's 'normalization process model' [13] as our guiding theoretical framework to help us understand the conditions necessary to support the introduction of computerised CBT both within the home and community locations. This practical model has been developed to assist the assessment and evaluation of complex interventions in health care and facilitates our use of the MRC framework, [9] enabling us to understand better how they can be embedded and integrated as routine elements of care. May suggests, for example, that a complex intervention such as computerised CBT is more likely to become part of routine clinical practice if it improves patient-professional interactions and confers an advantage on the organisation in terms of managing workload.

For the qualitative analysis, we will conduct semi-structured interviews with patients. Patients will be sampled based on their expressed preference for computerised CBT prior to randomisation, and whether they completed the course of computerised CBT or not. We expect to recruit between 36-40 patients, with the group reflecting a roughly equal balance of preference and completion. Patient interview topic guides will contain core questions developed from a literature review by Waller and Gilbody, [10] including computer literacy; changes in views about therapy pre and post-treatment; and views on using computerised CBT in future without consulting a health practitioner.Interviews will be held at the end of treatment and will be run throughout the duration of the data collection period.

We will also sample from patients who choose not to participate in the trial prior to randomization, if those patients indicate their primary reason for refusal is due to concerns over using computerised CBT. We expect to recruit between 8-10 patients in this group.

Health professional and managerial views on their experience of incorporating computerised CBT into primary care will be explored through individual semi-structured interviews with a purposive sample of GPs and practice managers. Topic guides will explore how this complex intervention is normalised within primary care. For GPs, this means, for example, that we will seek their views on potential changes to the doctor-patient relationship and for practice managers on issues of resources and risk. Interviews will continue in each group until data saturation is complete although we expect this may mean approximately 25 interviews with both GPs and managers.

3.1.3 Post trial modelling exercise: Decision modelling is increasingly used in the examination of clinical and cost effectiveness within health technology assessment; [15] often to enhance the relevance and applicability of randomised data and to allow additional economic and policy-related questions to be addressed (MRC complex interventions phase IV). Our post-trial modelling phase will enhance the results of the REEACT trial in the

following three ways: (1) costs and outcomes will be extrapolated beyond the 2-year time horizon of the trial to fully account for the longer term impact of the different interventions; (2) we will consider the results of the trial alongside other evidence that has emerged in the interim (computerised CBT is a rapidly evolving technology); (3) we will be able to provide an indirect comparison with therapist-led CBT.

Emergence and integration of additional interim evidence: Since the REEACT trial will not report longer term outcomes for four years, it is possible that the results of our trial will become supplanted by either new evidence or by the emergence of new products or costs. For example, if several trials of similar products emerge, it would be important to consider the results of the REEACT trial along with these other data. We will use meta-analysis and meta-regression to consider the clinical and cost effectiveness results from our own trial in comparison with other evidence. [16] Any new data can be entered as new input parameters and probabilistic sensitivity analyses within a simple decision model. [17]

Indirect comparison with face to face CBT: If computerised CBT were to be **clinically equivalent or not markedly inferior** to full therapist-delivered CBT, then there would be substantial benefits in adopting computerised CBT more widely within a stepped care framework; [8] given the potential lower costs, and increased accessibility. To date, there are no definitive trials of computerised CBT versus therapist-led CBT, and the ideal design to evaluate computerised CBT versus full CBT would be by adding a further treatment arm to the REEACT trial. However, in line with the research brief, we have proposed a three arm design (usual care vs free-to-use computerised CBT plus usual care vs pay-to-use computerised CBT plus usual care). We propose however, to address the comparative clinical and cost effectiveness of computerised CBT versus therapist CBT versus usual care alone using newly developed methods of **indirect and mixed treatment comparisons**. [18] This method uses a hierarchical Bayesian evidence synthesis to simulate head-to-head comparisons when these are not available directly from randomised evidence. This will be of substantial use to decision makers in implementing computerised CBT within a stepped care framework, when traditional models of face to face therapy remain the dominant model of CBT delivery.

3.2 Inclusion/Exclusion Criteria

3.2.1 Inclusion criteria: Our target population will be adult patients, aged 18 and above with depression who are not currently in receipt of computerised CBT or specialist psychological therapy. Our inclusion threshold will be a score of >=10 on the PHQ9 depression severity instrument. [19] This cut point is known to detect clinical depression (major depression) in a UK primary care population[20] with sensitivity = 91.7% and specificity = 78.3%. We will also include patients with either co-morbid physical illness or co-morbid non-psychotic functional disorders, such as anxiety. We will include both incident and prevalent cases. In line with the pragmatic nature of this trial, we will

reflect usual GP care and participants will be eligible to participate whether they are in receipt of antidepressant medication or not.

3.2.2 Exclusion criteria: We will exclude patients who are actively suicidal; suffering psychotic symptoms; depressed in the post-natal period; or have recently suffered bereavement. Patients with previous treatment experience of CBT will not be excluded. We will exclude cases of psychotic depression, since computerised therapy is not recommended within NICE guidance, [21] and are also unlikely to be recruited or randomised by general practitioners to receive computerised CBT, since they are unlikely to have sufficient equipoise in this case. We will also exclude patients who have alcohol or drug abuse as a primary diagnosis and patients who are not able to read and write in English.

3.3 Recruitment and Randomisation

We plan to use four main recruitment routes, as follows:

1. **GP-initiated recruitment.** When a GP identifies a potential trial participant, the GP will inform them about the trial. If the patient is immediately interested in participating, the GP will ask them to complete a ‘Permission for Release of Personal Details’ form that will include their contact details. The GP will also complete a referral form, stating that the patient matches the study criteria and give the patient a cover letter and a Patient Information Sheet. The GP or representative will then fax the referral form and the Permission for Release of Personal Details form to the local researcher. The researcher will then contact the patient to pre-screen for eligibility using the PHQ9 and, if appropriate, arrange a visit as soon as possible. Some patients may wish to consider participation over a longer period and the GP will give them the information pack and a letter that explains how to contact the research team. If the patient contacts the researcher he or she will then make an arrangement to visit the patient as soon as possible. When the researcher visits he or she will answer any questions about the trial, make sure that the patient has read the information sheet and obtain consent to be screened for eligibility (Part 1 of Patient Consent Form). The researcher will then check eligibility and obtain informed consent to participate in the study, if eligible (Part 2 of Patient Consent Form). The researcher will then take baseline measurements (Biographical Questionnaire, CIS-R, CORE-OM, SF-36 v2, EQ5D, adapted CSRI) and ascertain treatment preference. He or she will then contact the secure randomisation line to determine treatment allocation, whilst still with the patient. The researcher will then immediately inform the patient of the allocation and make arrangements to initiate computerised CBT if this is the allocation. A letter will also be faxed to the GP informing them of the outcome of the interview, together with the PHQ-9 score, if obtained. Researchers will also make arrangements to collect follow-up measurements after four months. This could mean arranging a follow-up visit or telephone call;

alternatively it may be preferable to the patient to receive and return the questionnaires by post. The researcher will also contact the patient shortly before the follow-up is due to confirm the arrangements, and re-arrange if necessary.

2. **Recruitment initiated by health professionals attached to a GP Practice**. We anticipate that practice-attached nurses and mental health professionals attached to GP Practices (‘primary care mental health workers’) will see people with depression who may be potential trial participants. They will be able to introduce the trial to the patient, give the patient relevant information and ask the patient to complete the ‘Permission to Contact’ form in the same way that a GP would. They will also be able to complete a referral form and fax both the ‘Permission to Contact’ form and the referral form to the local researcher. They will inform the patient’s GP that they have made a referral. The local researcher will proceed in a similar way to receiving a referral from a GP. The local researcher will let both the GP and the referrer know of the outcome of the referral.

3. **Record screening.** Where a GP agrees, we will ask practice staff to review patient records to identify any potential participants from previously recorded PHQ9 scores (collected in line with the Quality and Outcomes Framework). These prevalent cases will be contacted by post with the GP’s permission, or the GP can then consider the patient for the trial at their next appointment.

4. **Waiting room screening.** Where a GP practice agrees we will screen patients in the waiting room with a simple two question screening instrument. [23] If a patient screens positive for a possible depression, we will notify the GP immediately so that the GP can consider entering them into the trial.

Our trial will recruit in four centres – York and Gateshead (CI Gilbody), Bristol (investigators Araya and Kessler), Manchester (investigators Lovell, Gask, Bower, and Lester) and Sheffield (investigators, Barkham, Parry and Cooper). Each of these centres has an established recruiting network of practices. We have made a conservative assumption based on nationally available data that each GP with an average list size of 2,100 will see 2 new cases of depression per week. If we assume that GPs invite 25% of these incident patients to participate in the trial and that 25% of these patients meet our inclusion criteria and consent to participate, then each GP potentially contributes 10 patients during the two-year recruitment period. We have previously used waiting room screening methods and scrutiny of computerised records as described above to supplement this GP referral route, with an overall acceptance & randomisation rate of 25%. Our researchers will use active GP engagement with poorly recruiting practices and each of the investigators will provide participating practices with a series of mental health educational events to raise the profile of the REEACT study and as a participation

incentive. In line with recommendations by Peveler and colleagues from a HTA-funded primary care depression trial, we have included a service support cost to GPs and practices to compensate for additional time involved in recruitment. [24] A further GP incentive will be the use of the Quality and Outcomes Framework (QOF). [22] By participating in our trial, each practice will ensure that patients recruited into the trial also complete the PHQ9, - a validated assessment measure now incentivised for the Depression 2 indicator in the QOF.

Finally, to be sure that we will recruit to time and on target, we have enlisted the help of one of the Improving Access to Psychological Therapies (IAPT) programmes being coordinated by the Care Services Improvement Partnership (CSIP). This programme is assisting a number of PCT-based clinical sites to reconfigure their management of non-psychotic depression within a stepped care framework. Investigator Richards is an advisor to both the regional and national programmes. CSIP and the programme participants have agreed to allow eligible referred patients to be considered for inclusion in this trial. We will then follow the procedure for practice nurse initiated recruitment.

Subsequent to funding being secured, the REEACT trial has been adopted by the Mental Health Research Network (MHRN). New sites will be added and resources made available to help recruitment within NHS hub sites. The MHRN covers 60% of the population of England, and will be an invaluable resource for recruitment within the broad recruitment strategies specified in this protocol. The precise location of specific sites cannot be specified at this point in time.

Eligible participants who have consented to be in the trial will be randomised to treatment group using the computer-based York Trials Unit telephone randomisation service. In view of the large numbers enrolled in the study, further stratification by depression severity will not be needed. Stratification improves treatment precision in trials with less than 50 participants. However, for larger trials simple randomisation followed by analysis of covariance produces equally precise results[25] and eliminates any possible subversion associated with restricted randomisation methods.

3.4 Interventions

This is a pragmatic trial[26][27] and we will impose no restrictions on routine care, including the use of antidepressant drugs or the addition of drug treatment to computerised CBT in the intervention arms.

3.4.1 Experimental interventions: The intervention groups will each receive computerised therapy in addition to usual care. The control intervention will be usual GP care alone, with no specific encouragement to provide computerised CBT. Computerised therapy will be offered in one of three locations according to patient choice and local availability: (1) in the GP surgery (provided the patient's GP practice is able to provide a broadband-connected

computer and private room on a fixed weekly basis); (2) a central location close to the patients' own home e.g. in a psychotherapy department or a large GP practice, (where a stand alone computer in a private room operating on a weekly booking system will be provided); or (3) the patients' own homes (if they already have a computer and a broadband connection). This will maximise patient access and flexibility whilst respecting the importance of patient choice. All patients in the two intervention groups will receive support in the form of regular phone calls to encourage them to engage with the computerised therapy. We will record these phone calls in order to supervise the support worker. We will obtain informed consent to do this, but if participants do not consent to this, they can still participate in the trial.

Experimental group 1: Beating the Blues (©Ultrasis, http://www.ulltrasis.com) is an interactive, multimedia, computerised CBT package comprising a 15 minute introductory video followed by 8 therapy sessions of approximately 50 minutes duration each. There are homework exercises between the sessions. BtB has been shown to be effective in reducing symptoms of depression. [5] The usual purchase cost of this package is £800 per patient and it can be used on a stand-alone computer or via the Internet in the patient's own home.

Experimental group 2: MoodGYM (©ANU http://moodgym.anu.edu.au) is a free-to-use web-based CBT programme for depression developed and copyrighted at the Australian National University Centre for Mental Health Research. It consists of five interactive modules, which are made available sequentially weekly, with revision of all aspects of the programme in the sixth week. MoodGYM has been shown to be effective in reducing symptoms of depression. [6] MoodGYM is used in the UK with 20.5% of the registrants on MoodGYM being from the UK (Professor Kathy Griffiths - personal communication).

3.4.2 Control intervention: Participants allocated to the control condition will receive usual care by their general practitioner. In line with the overall pragmatic approach of the trial, we will replicate 'normal GP practice' by making no specific patient-level recommendation or requirement to alter usual care by participating in the trial. We will however remind GPs of the existence of NICE guidance on the management of depression, including the prescription of antidepressants, where this is indicated

3.5 Outcome Measures

3.5.1 Primary outcome measure: our primary outcome will be depression severity and symptomatology as measured by a validated self-report measure (the Patient Health Questionnaire-9) [19] at **four months**. The PHQ9 is a nine-item questionnaire, which records the core symptoms of depression. There are extensive US and non-US validation and sensitivity to change data. It has most recently been validated in a UK primary care population[20] and

has become the instrument of choice in UK primary care, [29] in the fulfilment of QOF routine depression measurement. It has the added advantage that it can be reliably administered over the phone. [30]

3.5.2: Secondary outcome measures: PHQ9 at 12 and 24 months; generic and global mental health (Clinical Outcomes in Routine Evaluation-Outcome Measure – CORE-OM); [31] Health Related Quality of Life (SF-36 v2); [32] health-state utility (EuroQol - EQ5D); adapted CSRI; Preference Questionnaire; each all at 4, 12 and 24 month. [33].

We will measure antidepressant use in all three arms to assess whether participants randomised to the CCBT arms are subject to differing prescription rates of antidepressants. We will do this by accessing the GP notes.

3.5.3: Outcome measures for primary care depression cohort: PHQ9; CORE-OM; a self-report measure of anxiety (GAD-7) [34], Health Related-Quality of Life (SF-12) [35]; EQ5D; adapted CSRI; ; each all at three, five, seven and ten years. We will also measure aspects of service use, concurrent physical health problems; and prescription of medication, by accessing GP notes.

4. Statistical Considerations

We will recruit a minimum of 690 patients with depression - 230 participants per arm. We need to know whether computerised CBT represents a useful addition to usual GP care, and whether the free to use computerised packages do not represent a poor second choice for patients. In this sense we will need to establish whether the clinical effectiveness of free to use computerised therapy is similar and NOT SUBSTANTIALLY INFERIOR to commercially produced CBT. Accordingly, we have powered our trial on the basis of non-inferiority, and will analyse our data in line with specific adjustments and recommendations that are needed in a non-inferiority trial. [36][37]

We have based our sample size on the usual care arm of our own recent primary care trial of collaborative care for depression, where the proportion of patients responding to usual care according to a SCID diagnosis (major depression, yes/no) in a usual care arm was in the region of 0.6 (similar rate found in a UK HTA trial of anti-depressants in primary care, [24] and US pragmatic depression trial. [368). We would regard a response rate not more than 0.15 below this rate as being acceptable, given the additional care options that are available to patients who do not initially respond to computerised CBT, within a stepped care framework. Our proposed sample size of 690 (230 participants per arm) will have in excess of 90% power to detect non-inferiority with a 5% probability. We have made an assessment of loss to follow up of 35% based upon our early recruitment in the trial, and by recruiting no fewer than 690 we will retain 80% power to detect non-inferiority.

In addition, our trial will also have 84% power to detect a difference of 0.15 between usual care arms and either of the computerised CBT arms using a conventional power analysis (alpha = 0.05; two sided)

5. Analysis

5.1 Statistical analysis of clinical data

We will analyse the data on an intention to treat basis. The primary outcome, depressed/not depressed (at four months) will be used in a logistic regression model to compare each of the computerised packages with usual care alone. The analysis will be adjusted for baseline depression severity (measured by PHQ9) and co-morbidity with anxiety (established by diagnosis using the CIS-R). [28] Odds ratios and the corresponding 95% confidence intervals will be presented for the two comparisons (BtB vs usual care and MoodGYM vs usual care). Two-sided 95% confidence intervals for the odds ratio will be calculated. Using this method, the experimental treatment is not inferior to the control treatment at a 5% level if the upper boundary is below our pre-specified margin of non-inferiority. [39]

For each outcome measure the number of non-responders will be calculated for each treatment group and response rates compared. Appropriate sensitivity analyses will be used to examine the effects of missing data on outcomes. All secondary analyses will be conducted using linear or logistic regression, depending on the outcome measure, adjusting for the same covariates as the primary analysis. The influence of preference will be ascertained by including this as a predictive co-variate in a planned sub-group analysis.

5.2 Analysis of economic data

The cost-effectiveness of computerised CBT will be evaluated in two phases. *Phase 1* will comprise a within-trial economic analysis using prospectively collected clinical and resource-use data within the trial, over a two-year time horizon. Individual patient-level data will be used to quantify costs during the study and quality of life will be assessed by the EuroQoL-5D (EQ-5D) questionnaire. *Phase 2* will address two related issues: (a) the need to extrapolate individual costs and quality of life data beyond the trial study period, and (b) the need to assess the cost effectiveness of the treatment strategies being investigated in the trial within the broader perspective of the NHS. Phase 2 of the study will, therefore, require the development of an economic model to (a) predict long term costs and effects and (b) synthesise available evidence regarding the effectiveness of alternative treatment strategies in order that the full range of possible treatment options and related evidence is evaluated in terms of cost-effectiveness. Overall cost-effectiveness for the 2 phases will be expressed in terms of the additional cost per quality-adjusted life-year (QALY) gained. Uncertainty in cost-effectiveness will be presented in terms of the probability that the alternative

forms of management are most cost-effective given a range of maximum values the NHS might be willing to pay for an additional QALY. [40]

Phase 1 Economic evaluation: Cost estimation under alternative treatment strategies will be done according to a two-stage process. The first stage is to measure resource use in physical units as used by trial patients. The second stage is to 'cost' these resource use data using prices or unit costs. Costs will be assessed from an NHS and Personal Social Service perspective. A separate analysis of costs from a wider perspective will also be undertaken, reflecting the societal perspective of the recent Layard report. [7] The wider social costs will be presented separately to avoid double counting with the QALY measure. Resource utilisation will be assessed from case records and patient self-report using an adapted version of the Client Service Receipt Inventory (CSRI). [41] The study will aim to estimate representative national unit costs. Intervention costs will be based on delivery costs within the trial and include supervision and appropriate capital costs.

In this study the main outcome for the cost-effectiveness analysis will be the QALY, assessed using EQ-5D, [33] which consists of five health dimensions: mobility, self-care, usual activities, pain/discomfort and anxiety/depression. Each dimension has three levels of severity (no problems, moderate problems and severe problems) that generate 245 unique health states into which a patient can be classified. In addition to providing a description of the health states, the EQ-5D also provides a single preference weight (also described as a utility or value) for each health state. The use of generic, validated quality of life measures and general population weights in the application of cost-effectiveness analysis has been recommended for policy level decision-making and now form part of the Reference Case for cost-effectiveness studies submitted to NICE.

Phase 2 Economic evaluation: The trial data will provide estimates of costs and effects that initially follow the clinical outcome data. However, cost-effectiveness analysis should adopt a time-horizon over which the costs and benefits of alternative treatments may vary. To fully account for the longer-term costs and benefits of the alternative treatments it will be necessary to extrapolate beyond the trial period. Statistical and decision-modelling methods will be used to undertake extrapolation, which will apply to costs as well as effects. This will take the form of a cohort state transition model which will reflect, for each treatment being evaluated: (i) the intervention costs of the study treatments; (ii) mortality rates and impact on quality of life of the therapies; (iii) rates of response and relapse over time; and (iv) costs and quality of life of response and relapse. The model will estimate long-term quality-adjusted survival and costs for each intervention.

In order to provide a policy-relevant analysis to provide an input into decision-making, it is important for Phase 2 of the project to draw evidence from sources other than just the REEACT trial. In particular, we will seek to incorporate published data from all relevant trials relating to the use of computerised CBT into the long-term model. Furthermore we will seek to

combine these data with other trial data to facilitate a direct comparison of the cost-effectiveness of computerised CBT compared to therapist-led CBT.

To inform future research priorities in the NHS, the model will also be used to undertake analyses of the expected value of information. [42] Bayesian value of information analysis can used to determine the expected costs of decision uncertainty predicted by the model and the maximum value that can be placed on additional research aimed at reducing this uncertainty. This analysis will be used as the basis to inform policy decisions relating to future research priorities in this area.

5.3 Qualitative analysis of process data

All focus groups and semi-structured interviews will be audio taped and fully transcribed. Transcripts and notes will be read and re-read independently by two of the research team (Lester and Gask). The data will then be organised into initial codes and higher codes that provide insight into emergent themes. Key concepts and categories will be identified using an open coding method by deconstructing each interview sentence by sentence. Main categories will then be compared across interviews / focus groups and reintegrated into common themes, The focus group data analysis will also seek issues with strong group-to-group validation and "sensitive moments" within group interactions that indicated difficult but important issues. [43]Reliability will be enhanced by identifying issues that are consistent between groups and validated using 'sensitive moments' within focus group interactions that indicate difficult but important issues. [44] A computer software package (QSR NVivo) will be used to manage the data and to increase the transparency of the analysis. Deviant cases will be actively sought throughout the analysis and emerging ideas and themes modified in response. [45]

6. Ethical Issues

We are aware that people with depression represent a vulnerable group. However, we do not anticipate any major ethical issues with the proposed study since both experimental treatments are in use in the NHS or are recommended in recent guidance issued by the National Institute of Clinical Excellence. [21] However, computerised therapy is not *routinely* available in primary care at the current time (i.e. it is subject to constrained access and geographical variations in availability). We therefore have some concern that there may be insufficient individual equipoise amongst GPs and patients to consider randomisation to a treatment (computerised CBT) with which they have little experience. We have addressed this issue in the pragmatic design of our study. Patients allocated to computerised therapy will still receive usual care (including antidepressants where the GP decides this is necessary). We will report antidepressant use as an outcome of interest. The issue of patient preference will be ascertained at study entry and we will carry out a planned subgroup analysis. We will explore the issue of clinician equipoise in qualitative interviews.

6.1 Risks and anticipated benefits for trial participants and society

All participants will receive usual GP care, and therefore no treatment will be withheld by participating in this trial. This trial may in fact benefit individual participants, since computerised therapy is not routinely available despite NICE recommendations. By participating in this trial, patients will also receive a more intensive level of monitoring than that normally received in primary care. Patients who fail to respond to computerised therapy; deteriorate rapidly or become suicidal will be more readily identified and directed to appropriate care.

6.2 Informing potential participants of possible benefits and known risks

The patient information leaflets will provide potential participants with information about the possible benefits and any known risks of taking part in the trial. Participants will be given the opportunity to discuss this issue with either their GP or trial coordinator prior to consenting to participate. The trial coordinator will inform the participant if new information comes to light that may affect the participant's willingness to participate in the trial.

6.3 Obtaining informed consent from participants

Potential participants will receive an information pack about the trial. The pack will contain an invitation letter and patient information leaflet., The researcher will assess them for eligibility and then discuss the trial and answer any questions. Written informed consent will then be obtained prior to the patient being randomised.

6.4 Proposed time period of retention of relevant trial documentation

All data will be stored for a minimum of 20 years after the end of final analysis of the study and will be accessed by the Trial Statistician. All paper records will be stored in a secure storage facilities. Personal identifiable paper records will be stored separate from anonymised paper records. All electronic records will be stored on a password protected server within York Trials Unit and the University of Manchester. All contact information will be destroyed securely immediately at the end of the trial.

7. Service User Involvement

Service-user input into the design, conduct and dissemination of the REEACT trial comes from Nicky Lidbetter, who is a named co-applicant. Nicky is both a mental health service user and is involved in the delivery and implementation of computerised self-help packages within NHS primary care. Nicky has been a non-executive director of Manchester Mental Health & Social Care NHS Trust since 2002 where she had responsibility for chairing the Trust's Research & Development Committee. Very recently, Nicky has established

the first user-led primary care mental health service in Central Manchester and is soon to launch a new user-led computerised Cognitive Behavioural Therapy project in conjunction with Manchester PCT and CSIP North West. Service users will also be invited to join the trial steering group and we will follow good practice in terms of ensuring their ability to contribute to discussions. We will also work with our service user applicant to ensure that our dissemination strategies are inclusive and accessible to people who use services.

8. Research Governance

The trial will be conducted to protect the human rights and dignity of the participant as reflected in the 1996 version of the Helsinki Declaration. Patients will not receive any financial inducement to participate. In order to protect the trial participants the following provisions will be made/upheld; the trial has been designed to minimise pain, discomfort and fear and any foreseeable risk in relation to the treatments involved, the explicit wishes of the participant will be respected including the right to withdraw from the trial at any time, the interest of the patient will prevail over those of science and society, provision will be made for indemnity by the investigator and sponsor and a contact name for further information will be provided.

8.1 Trial sponsorship

The University of York have agreed to act as sponsors for the trial.

8.2 Monitoring and reporting adverse events

We will have no influence on the prescription of medications by the GP for participants in this trial. We propose no experimental manipulation to directly influence choice or dose of medication. This trial will not therefore be subject to any additional restrictions, such as being Clinical Trial of an Investigational Medicinal Product (CTIMP). We have sought and received guidance on this from the MHRA in October 2008

Monitoring: Researchers will ask participants about any health-related adverse events at each follow-up visit (4, 12 and 24 months). For any events that are judged to be serious, the researcher will complete a Serious Adverse Event/Reaction (SAE/R) form (Appendix 1). We will also ask GPs involved in the trial to complete one of these forms if they identify that a trial participant has experienced a serious adverse event. For non-serious adverse events (NSAEs), the researcher will complete a NSAE form.

Reporting: Reporting of SAE/Rs should take place as soon as the GP or local researcher becomes aware of the event. GPs will be asked to immediately fax SAE/R forms to the local trial centre. The local researcher/PI should immediately report all SAE/Rs to the Trial Manager by telephone. The SAE/R form should be faxed to the Trial Manager within 48 hours. A copy of

the form should be kept by the local trial centre. If a SAE/R is reported and the paperwork completed by someone other than the PI, it is important that this person discusses the event with the PI as soon as possible.

The Trial Manager will then inform the Chief Investigator and at least 2 members of the Trial Management Group will decide whether the event is related to the trial treatment. The DMEC and TSC will immediately see all SAE/Rs thought by the Trial Management Group to be treatment-related.

All SAE/Rs will be reported to the main REC within 15 days of the CI becoming aware of the event where the event is related to administration of any of the research procedures and is unexpected.

Local researchers should inform the Trial Manager of any NSAEs within 5 days of becoming aware of the event. He or she should also complete a NSAE form which should be sent to the Trial Manager and a copy kept at the local trial centre. The CI and Trial Manager will review NSAE forms to check the assessment of seriousness and of relatedness to the treatment.

The DMEC/TSC will review the following at their next scheduled meeting

- SAEs not thought to be treatment related by the Trial Management Group
- NSAEs thought to be related to the treatment
- NSAEs thought to be unrelated to the treatment

Suicide

Inherent in the nature of the condition under scrutiny (depression) is the risk of suicide and deliberate self-harm. All participants will be subject to usual GP care, and the primary care physician will be responsible for the day to day management of depression – and will ultimately be responsible for all patient-level treatment/management decisions – including prescribing, referral and assessment of risk. This arrangement is made clear to all clinicians who refer patients to this trial. The pragmatic nature of this trial means that we will not seek to influence this arrangement. However, we will follow good clinical practice in monitoring for suicide risk during all researcher encounters with trial participants. Where any risk to patients due to expressed thoughts of self-harm is encountered, we will follow the trial suicide protocol (see Appendix II).

9. Study Organisational Structure

9.1 Trial Steering Committee (TSC)

A TSC will be set up and will include an independent chair and at least two other independent members, including a service user, along with the chief investigator and the other study investigators. They will meet at least annually.

9.2 Data Monitoring and Ethics Committee (DMEC)

A DMEC will be set up and will include an independent statistician and mental health professional. The role of the DMEC is to immediately see all serious adverse events thought to be treatment related and look at the outcome data from an ethical standpoint. They will meet at least annually.

The membership of our TSC and DMEC is detailed in appendix III.

9.3 Trial management

The Trial Management Group will consist of the CI, other investigators, Trial Manager, Data Manager and Trial Statistician and will consider day-to-day management issues and the overall progress of the trial They will meet quarterly.

The York-based trial manager will be responsible for the day-to-day running of the trial, obtaining ethical and research and development approval, designing trial documentation, recruitment of GPs and participants and trial centre co-ordination, collection of data, assisting the statistician clean and analyse data, writing the initial draft of research papers and disseminating the study's findings. The trial secretary will assist the trial co-ordinator with these tasks whilst the data manager will validate and manage the data prior to analysis. The trial statistician will be responsible for cleaning the data, conducting the statistical analysis and sending the data to the DMEC. Professors Lester and Gask will be responsible for the process evaluation element of the trial. The Chief Investigator will be in charge of the overall management of the trial.

Each trial site (York, Manchester, Sheffield, Bristol) will have a research fellow/trial coordinator to facilitate recruitment; liaise with GPs; to introduce and monitor computerised therapy; and to ensure patient follow up and outcome assessment. They will be aided by a part time secretary/admin worker who will co-ordinate patient computer appointments. Clinical supervision and GP liaison will be undertaken by a research active clinician with specific experience in CBT (York - Gilbody & Richards; Manchester - Lovell and Gask; Sheffield - Parry; Bristol - Araya and Kessler). For years 3 & 4, site research fellows will ensure patient follow up and scrutiny of GP records to establish service utilisation and antidepressant prescriptions and conduct post-intervention qualitative interviews. The research fellow in Manchester will, after appropriate training, also assist Professors Lester and Gask in undertaking the focus groups with patients and in interviewing GPs and practice managers. The economic analysis will require the specialist input of Dr Palmer and Professor Sculpher (main analysis in year 5).

10. Project Timetable and Milestones

**1st April 2008**	Apply for ethics, research and development (R & D) approval for all sites as required and for adoption onto the MHRN.
**May 2009-May 2011**	Start patient recruitment at York, Manchester, Bristol and Sheffield, plus sites from MHRN/CSIP collaborators as soon as Ethics/ R & D approval are received for a 24-month period.
**May 2013 – End of Trial**	Final follow-up interview with last participant.
**May 2013 to end October 2013**	Data cleaning and statistical analysis and writing up study findings.
**Jan 2013 to 2021**	Extended follow-up interviews with consenting trial participants (Primary Care Depression Cohort)

11. Statement of Indemnity

Normal NHS Indemnity procedures will apply. The University of York will also provide relevant cover.

12. Dissemination

We will publish papers relating to this trial that will include (as a minimum) the results of the clinical and cost effectiveness comparisons and the results of the qualitative analysis. Professor Gilbody is an editor of the Cochrane Depression, Anxiety and Neurosis Group and can thus ensure that the results of this trial are incorporated in relevant Cochrane reviews. We will also publish in professional journals to ensure that clinicians have prompt access to our findings. We will produce a short summary of the results that can be distributed to all trial participants, including patients and GPs, as well as relevant patient and other interest groups. Finally, we will aim to ensure coverage of our findings in the wider media by issuing a press release. This will serve to bring the public and clinicians' attention to our findings.

13. References

1. Roth, A. and P. Fonagy, *What works for whom?: a critical review of psychotherapy research*. 1996, New York: Guilford Press.
2. Department of Health, *A review of strategic policy on NHS psychotherapy services in England*. 1996, London: HMSO.
3. Lovell, K. and D. Richards, *Multiple Access Points and Levels of Entry (MAPLE): Ensuring choice, accessibility and equity for CBT services.* Behavioural and Cognitive Psychotherapy, 2000. **28**: p. 379-391.
4. Kaltenthaler, E., et al., *Computerised cognitive behaviour therapy for depression and anxiety update: a systematic review and economic evaluation.* Health Technology Assessment, 2006. **10**: p. 33.
5. Proudfoot, J., et al., *Computerised, interactive, multimedia cognitive behavioural therapy for anxiety and depression in general practice.* Psychological Medicine, 2003. **33**: p. 217-227.

6. Christensen, H., K.M. Griffiths, and A.F. Jorm, *Delivering interventions for depression by using the internet: randomised controlled trial.* BMJ, 2004. **328**: p. 265.
7. Layard, R., *The case for psychological treatment centres.* BMJ, 2006. **332**: p. 1030-2.
8. Bower, P. and S. Gilbody, *Stepped care in psychological therapies: access, effectiveness and efficiency. Narrative literature review.* Br J Psychiatry, 2005. **186**: p. 11-7.
9. Campbell, M., et al., *Framework for design and evaluation of complex interventions to improve health.* British Medical Journal, 2000. **321**: p. 694-696.
10. Waller, R. and S. Gilbody, *Barriers to the uptake of computerised cognitive behavioural therapy: A systematic review of the quantitative and qualitative evidence.* Psychological Medicine, 2008. **In press**.
11. Torgerson, D. and J.K. Moffett, *Patient Preference and Validity of Randomized Controlled Trials.* JAMA, 2005. **294**(1): p. 41-a-42.
12. Howard, L. and G. Thornicroft, *Patient preference randomised controlled trials in mental health research.* Br. J. Psychiatry, 2006. **188**: p. 303 - 304.
13. May, C.R., *A rational model for assessing and evaluating complex interventions in health care.* BMC Health Services Research, 2006. **6**: p. 86.
14. Morgan, D.L., *Focus groups as qualitative research*. 1997, London: Sage.
15. Claxton, K., M. Sculpher, and M. Drummond, *A rational framework for decision making by the National Institute For Clinical Excellence (NICE).* Lancet, 2002. **360**: p. 711-5.
16. Thompson, S.G. and J.P. Higgins, *How should meta-regression analyses be undertaken and interpreted?* Stat Med, 2002. **21**: p. 1559-73.
17. Sculpher, M., M. Drummond, and M.J. Buxton, *The iterative use of economic evaluation as part of the process of health technology assessment.* J Health Serv Res Policy, 1997. **2**: p. 26-30.
18. Caldwell, D.M., A.E. Ades, and J.P.T. Higgins, *Simultaneous comparison of multiple treatments: combining direct and indirect evidence.* BMJ, 2005. **331**: p. 897 - 900.
19. Spitzer, R.L., K. Kroenke, and J.B.W. Williams, *Validation and utility of a self-report version of PRIME-MD: the PHQ primary care study. Primary Care Evaluation of Mental Disorders. Patient Health Questionnaire.* JAMA, 1999. **282**: p. 1737-44.
20. Gilbody, S., D. Richards, and M. Barkham, *Diagnosing depression in primary care using self-completed instruments: a UK validation of the PHQ9 and CORE-OM.* British Journal of General Practice, 2007. **57**(541): p. 65-652.
21. National Institute for Clinical Excellence, *Depression: core interventions in the management of depression in primary and secondary care*. 2004, London: HMSO.
22. BMA and NHS Employers, *Revisions to the GMS contract, 2006/7. Delivering investment in General Practice*. 2006, London: British Medical Association.
23. Whooley, M.A., et al., *Case finding instruments for depression two questions as good as many.* J Gen Intern Med, 1997. **12**: p. 439 -45.
24. Peveler, R., et al., *A randomised controlled trial to compare the cost-effectiveness of tricyclic antidepressants, selective serotonin reuptake inhibitors and lofepramine.* Health Technol Assess, 2005. **9(16)**: p. 1-134.
25. Rosenberger, W.F. and J.M. Lachin, *Randomization in clinical trials: theory and practice*. 2002, New York: Wiley & Sons.
26. Schwartz, D. and J. Lelloch, *Explanatory and pragmatic attitudes in therapeutic trials.* Journal of Chronic Diseases, 1967. **20**: p. 637-648.
27. Hotopf, M., R. Churchill, and G. Lewis, *Pragmatic randomised trials in psychiatry.* British Journal of Psychiatry, 1999. **175**: p. 217-223.
28. Lewis, G., et al., *Measuring psychiatric disorder in the community: a standardized assessment for use by lay interviewers.* Psychological Medicine, 1992. **22**: p. 465-486.
29. Fletcher, J., et al., *Enhanced services specification for depression under the new GP contract*. 2004, Manchester: NIMHE Northwest.
30. Pinto-Meza, A., et al., *Assessing depression in primary care with the PHQ-9: can it be carried out over the telephone?* J Gen Intern Med, 2005. **20**: p. 738-42.
31. Barkham, M., et al., *Service profiling and outcomes benchmarking using the CORE-OM: Towards practice-based evidence in the psychological therapies.* Journal of Consulting and Clinical Psychology, 2001. **69**: p. 184-196.

32. Ware, J.E., M. Kosinski, and J.E. Dewey, *How to Score Version Two of the SF-36 Health Survey*. 2000, Lincoln, RI: QualityMetric Incorporated.
33. EuroQol Group, *Euroqol - a new facility for the measurement of health related quality of life.* Health Policy, 1990. **16**: p. 199-208.
34. Spitzer, R., Kroenke, K., Williams, J. and Lowe, B. *A brief measure for assessing generalised anxiety disorder: The GAD:7*. Archives of Internal Medicine. 2006. **166**: p. 1092-1097.
35. Ware, J.E., Kosinski, M. and Keller, S.D. *A 12-Item Short-Form Health Survey: Construction of scales and preliminary tests of reliability and validity*. Medical Care. 1996. **34(3)**: p. 220-233.
36. Jones, B., et al., *Trials to assess equivalence: the importance of rigorous methods*. 1996, Br Med Assoc.
37. Piaggio, G., et al., *Reporting of Noninferiority and Equivalence Randomized TrialsAn Extension of the CONSORT Statement*. 2006, Am Med Assoc.
38. Simon, G., E. Wagner, and M. Vonkorff, *Cost-effectiveness comparisons using "real world" randomized trials: the case of new antidepressant drugs.* Journal of Clinical Epidemiology, 1995. **48**(3): p. 363-73.
39. Hollis, S. and F. Campbell, *What is meant by intention to treat analysis? Survey of published randomised controlled trials.* BMJ, 1999. **319**(7211): p. 670-674.
40. Fenwick, L., K. Claxton, and M. Schulpher, *Representing uncertainty: the role of cost-effectiveness acceptability curves.* Health Economics, 2002. **10**: p. 779-787.
41. Knapp, M. and J. Beecham, *Costing mental health services.* Psychological Medicine, 1990. **20**: p. 893-908.
42. Claxton, K., et al., *A pilot study on the use of decision theory and value of information analysis as part of the NHS Health Technology Assessment programme.* Health Technol Assess, 2004. **8**(31): p. 1-103.
43. Barbour, R. and J. Kitzinger, *Developing focus group research. Politics, theory and practice*. 1999, London: Sage.
44. Barbour, R., *Checklist for improving rigour in qualitative research: case of the tail wagging the dog?* BMJ, 2001. **322**: p. 115-7.
45. Silverman, D., *Interpreting qualitative data. Methods for analysing talk, text and interaction*. 1997, London: Sage.

Appendix I

Serious Adverse Event/ Reaction Form

reeact

THE UNIVERSITY of York

SERIOUS ADVERSE EVENT/REACTION FORM
Computerised Cognitive Behaviour Therapy for Depression

REEACT is required to report quickly to our main Research Ethics Committee **any serious adverse events that may be related to the trial treatment**. We also need to know about serious adverse events that are not related to the trial treatment. To enable us to do this, please let us know **as soon as possible** of any serious events experienced by trial participants so that we can judge if they are trial related.. Please complete this form as fully as you can and fax to your local REEACT centre on xxxxx.

Serious events/reactions are defined as fatal, life-threatening, resulting in persistent or significant disability or incapacity, resulting in or prolonging hospitalisation, resulting in a congenital anomaly or birth defect, or those which are deemed by the reporter as medically significant.

Patient Details

REEACT ID Number: GP Patient Number:

Sex (please circle): **M / F** Date of Birth:

Patient Initials Weight (kgs) Height (cms)

Event Details

Please describe the event, any treatment given and the outcome

Date event started………………………… Date event stopped (if applicable)…………………...
Please indicate **why** you consider this event to be serious (please tick all that apply)

Patient died ☐ Involved inpatient hospitalisation ☐

Life-threatening ☐ Involved persistent or significant disability or incapacity ☐

Resulted in a congenital anomaly or birth defect ☐

Relationship of Event to Treatment (tick one box only)

Unrelated ☐ Unlikely to be related ☐ Possibly related ☐ Probably related ☐ Definitely related ☐ Not able to assess if related ☐

Your Details

Name, position and professional address

…………………………………………………………………………………………………….

…………………………………………………………………………………………………….

…………………………………………………………………………………………………….

Tel No: …………………………….   Profession (Specialty) ……………………………………..

Signature .... Date……………………………………………

Please FAX this form to REEACT on XXXXXXX

Thank You

Appendix II

Suicide Protocol

If at any time you believe that there is a significant suicide risk with a patient who is participating in the study that has not been communicated to their GP, you must contact Professor Simon Gilbody (an honorary consultant psychiatrist), or the relevant designated centre psychiatrist or psychologist in Bristol, Sheffield or Manchester, if Professor Gilbody is not available.

Professor Gilbody, or designated psychologist/psychiatrist, will then assess the patient and if he / she believes it necessary and if there is a significant risk, he / she will notify the patient's GP **with or without** their consent. However Professor Gilbody or the designated psychologist/psychiatrist would contact the GP without first assessing the patient for himself/herself if the situation was urgent, again with or without the patient's consent.

The PHQ9 questionnaire asks if the patient has had **"Thoughts that you would be better off dead or of hurting yourself in some way" (Question 9).** If participants indicate a response of **3** for this item, then you should ask whether the patient has talked to their GP about these feelings**. If the patient has spoken of these thoughts to their GP then no action is required.**

If not, you should ask the patient whether it is OK for you to contact their GP and inform them of the situation. If the patient refuses, contact Professor Gilbody, or the relevant designated psychiatrist/psychologist. If the patient agrees, you should immediately get in touch with the appropriate GP.

If unable to contact Professor Gilbody or any of the designated centre psychologists/psychologists, contact the Trial Co-ordinator, Dr Liz Littlewood, or any other of the Co-investigators who will advise further.

You should follow the same procedure if the results of the CIS-R indicate that **the patient has had suicidal plans in the past week.**

The diagram below illustrates this procedure.

Please also complete the attached Suicidal Intent Form, if the patient agrees to you contacting their GP, and inform the Trial Co-ordinator. If relevant, Professor Gilbody or the relevant designated centre psychiatrist/psychologist should also complete the Suicidal Intent Form: Psychiatrist/Psychologist. These forms should then be stored with the patient's trial records.

Suicide Risk Identified on a Postal Questionnaire

Some patients may choose to receive and return the follow-up questionnaires by post at any of the follow-up points (4 months, 12 months or 24 months). If you receive a PHQ9 in which the patient has indicated a score of **3** for Question 9, you will also need to follow the suicide protocol. Contact the patient by telephone and say that you are concerned by their response to this

question. Ask if they have discussed these feelings with their GP. **If the patient has spoken of these thoughts to their GP then no action is required.** If not, you should ask the patient whether it is OK for you to contact their GP and inform them of the situation. If the patient refuses, contact Professor Gilbody, or the relevant designated psychiatrist/psychologist. If the patient agrees, you should immediately get in touch with the appropriate GP.

If unable to contact Professor Gilbody or any of the designated centre psychologists/psychologists, contact the Trial Co-ordinator, Dr Liz Littlewood, or any other of the Co-investigators who will advise further.

If any other written responses on the questionnaires give you cause for concern, raise this with Professor Gilbody, or the relevant designated psychiatrist/psychologist.

If you are unable to contact the patient within 24 hours, contact the patient's GP. Inform the GP of the patient's questionnaire response and that you have been unable to contact the patient to assess the situation further.

At this point also check that the patient's contact telephone number is correct. It may be that we have an out of date telephone number. If an alternative telephone number is provided and the GP agrees, attempt to contact the patient again.

If still unable to contact the patient or if no alternative contact number is available confirm with the GP that they will follow up with the patient as they feel appropriate based on their clinical knowledge of the patient.

Inform Professor Gilbody, or the relevant designated psychiatrist/psychologist of the patient's questionnaire response and details of contact with the GP.

Complete the appropriate forms as for a face-to-face/ telephone interview.

If, at any time, you have any concerns surrounding this, speak to Professor Gilbody or the designated centre psychiatrist/psychologist.

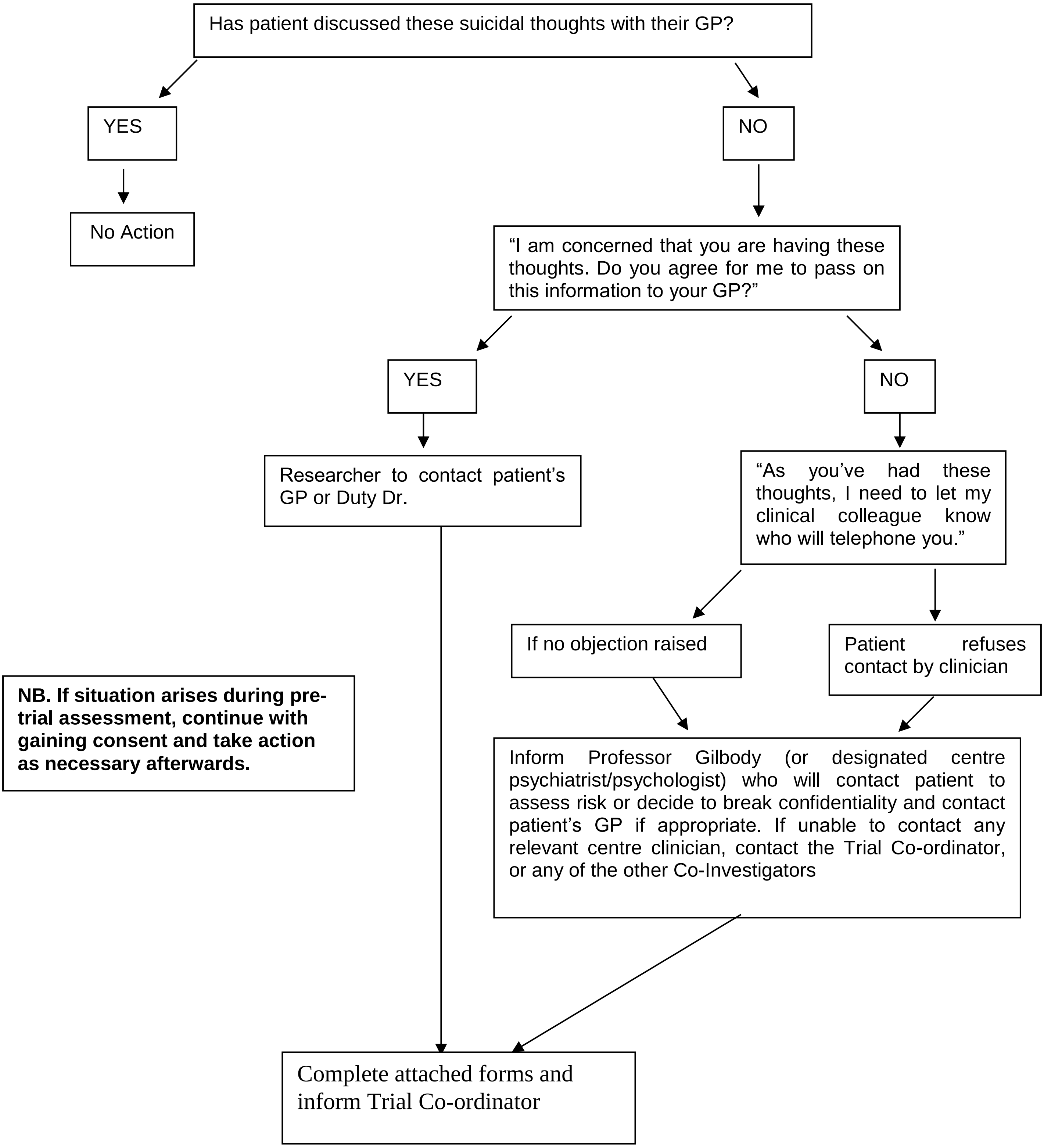
Has patient discussed these suicidal thoughts with their GP?
YES
NO
No Action
"I am concerned that you are having these thoughts. Do you agree for me to pass on this information to your GP?"
YES
NO
Researcher to contact patient's GP or Duty Dr.
"As you've had these thoughts, I need to let my clinical colleague know who will telephone you."
If no objection raised
Patient refuses contact by clinician
NB. If situation arises during pre-trial assessment, continue with gaining consent and take action as necessary afterwards.
Inform Professor Gilbody (or designated centre psychiatrist/psychologist) who will contact patient to assess risk or decide to break confidentiality and contact patient's GP if appropriate. If unable to contact any relevant centre clinician, contact the Trial Co-ordinator, or any of the other Co-Investigators
Complete attached forms and inform Trial Co-ordinator

SUICIDAL INTENT FORM

The patient below has shown thoughts of suicidal intent on the PHQ9 or CIS-R and has agreed for their GP to be contacted by the researcher

Name of participant: ……………………………………………

REEACT Participant ID: ……………………………

Suicidal intent revealed on PHQ9 ☐ CIS-R ☐

Action taken

Date GP contacted: ……………………

Name of GP contacted: ………………………………

Outcome of contact with GP/Comments

………………………………………………………………………………………

………………………………………………………………………………………

………………………………………………………………………………………

………………………………………………………………………………………

………………………………………………………………………………………

………………………………………………………………………………………

……………………………………………………………………………………..

SUICIDAL INTENT FORM: PSYCHIATRIST/PSYCHOLOGIST

Name of participant: ……………………………………………

REEACT Participant ID: ……………………………

Name of Psychiatrist/Psychologist notified: ………………………….

Date notified: …………………………

Action taken

Patient contacted: Yes ☐ No ☐

GP contacted: Yes ☐ No ☐

If Yes, GP contacted with patient's consent

Yes ☐ No ☐

Name of GP contacted: ………………………………

Date of contact: ……………………..

Comments

……………………………………………………………………………………

……………………………………………………………………………………

……………………………………………………………………………………

……………………………………………………………………………………

……………………………………………………………………………………

……………………………………………………………………………………

…………………………………………………………………………………..

Appendix III

DMEC and TSC membership

REEACT Trial Steering Committee

Professor Joe Reilly (Chair): Clinical Director for R&D (Tees, Esk and Wear Valleys NHS Trust), Director, Mental Health Research Centre (Durham University), Deputy Lead (Mental Health Research Network North East Hub)
j.g.reilly@durham.ac.uk

Dr Mike Slade: Reader in Health Services Research, Head of Section of Community Mental Health, Consultant Clinical
Psychologist in Rehabilitation (Institute of Psychiatry)
m.slade@iop.kcl.ac.uk

Professor Shirley Reynolds: Professor of Clinical Psychology (University of East Anglia)
S.Reynolds@uea.ac.uk

Professor Robbie Foy: Professor of Primary Care (University of Leeds)
r.foy@leeds.ac.uk

Mr Nic Seccombe: (Anxiety UK) – service user representative
cCBT Manager
SELF HELP SERVICES
Zion Community Centre
339 Stretford Road
Hulme, Manchester, M15 4ZY
ccbt@selfhelpservices.org.uk

Mrs Lina Gega: Lecturer in Mental Health (University of East Anglia)
L.Gega@uea.ac.uk

PLUS members of the REEACT co-investigators

REEACT Data Monitoring and Ethics Committee

Professor Mike Lucock (Chair): Professor of Clinical Psychology,,University of Huddersfield.
m.lucock@hud.ac.uk

Dr Chris Roberts: Reader in Biostatistics,University of Manchester.
Chris.roberts@manchester.ac.uk

Dr Paul Blenkiron: Consultant Psychiatrist & Cognitive Therapist (North Yorkshire and York PCT)
Paul.blenkiron@nyypct.nhs.uk
