# Supplementary material for: Computerised cognitive behaviour therapy (cCBT) as treatment for depression in primary care (REEACT trial): large scale pragmatic randomised controlled trial
Source: BMJ. 2015 Nov 11;351:h5627. doi: 10.1136/bmj.h5627 (PMC4641883; doi:10.1136/bmj.h5627)
Supplement: Supplementary file 2 [file gils026474.ww2_default.pdf]

Computerised Cognitive Behaviour Therapy for Depression

Patient Information Sheet

We would like to invite you to take part in a research study. Before you decide it is important for you to understand why the research is being done and what it would involve for you. Please take time to read the following information carefully. Feel free to discuss the study with your family, friends or GP.

- Part 1 tells you the purpose of this study and what will happen to you if you take part.
- Part 2 gives you more detailed information about the way in which the study will be run.

Ask us if there is anything that is not clear or if you would like more information. Our contact details can be found on Page 8. Take time to decide whether or not you wish to take part.

Part 1: General Information

What is the purpose of the study?

Depression causes misery to many people and is a major health problem in the UK. The majority of people with depression receive care from their GP, and never really see a specialist. However, lots of people experiencing depression would also like to receive a “talking treatment” (counselling or psychotherapy). Cognitive Behaviour Therapy (CBT) seems to be the most effective type of talking treatment for depression but is not always immediately available in the NHS.

Recently, therapists have developed a form of CBT that can be delivered by computer, which might make it easier to access this form of treatment. We call this “computerised CBT”. There are different computerised packages, which can be used in your own home or in your GP practice. At the moment we do not yet know which of these works best for NHS patients with depression.

Computerised CBT is recommended by the National Institute for Clinical Excellence (NICE) for people experiencing depression, but we need more information about how effective it is. We would also like to know how different computerised CBT packages compare. This is important for the NHS to know, when deciding whether to offer this treatment in General Practice.

The purpose of this study is to compare two commonly used CBT packages to see if there are any additional benefits of offering this treatment to the care that people already receive from their General Practitioner.

Why have I been invited?

Your GP is taking part in this study and has identified you as suffering from depression. We are planning to recruit **690** people with depression to take part in the study.

Do I have to take part?

It is up to you to decide. We will describe the study and go through this information sheet with you. We will then ask you to sign a consent form to show you have agreed to take part. You are free to withdraw at any time, without giving a reason. This would not affect the standard or type of care you receive.

What will happen to me if I take part?

This study is a randomised controlled trial. Sometimes we don't know which way of treating patients is best. To find out, we need to compare different treatments. We put patients into groups and give each group a different treatment. The results are compared to see if one is better, or if they are all equally as effective. To try to make sure the groups are the same to start with, each patient is put into a group by chance (randomly). We will be putting patients into one of three groups (usual GP care alone, or one of two forms of computerised CBT plus usual GP care). A third of all study participants (**230**) will go into each group. You therefore have a one-in-three chance of going into a particular group.

Firstly we will ask you some brief questions over the telephone to see if you are likely to be eligible for the study. We will then interview you at a venue you choose. We will ask for your consent to ask you some questions to check that you are definitely eligible for the study. If you are eligible we will then ask for your consent to enter you into the study. We will then ask you to fill in some questionnaires (we can help you with this if necessary) and we will make a telephone call to find out which treatment group to put you in. We will then let you know how to access your treatment. This first interview will last about an hour.

After the initial interview patients in the usual GP care group will continue to receive care for their depression from their GP. Patients receiving one form of computerised CBT (*Beating the Blues*) will attend 8 sessions of therapy at a convenient location. Each session will last about an hour and there will be some 'homework' to be done between sessions. If patients have a home computer and Internet connection, these sessions can be done online at

home. Patients receiving the other form of computerised CBT (*MoodGYM*) will attend 6 sessions of therapy at a convenient location. Each session will last about an hour and there will be some 'homework' to be done between sessions. Again if you have a computer and Internet connection, these sessions can be done online at home.

If you receive computerised CBT, we will also telephone you several times over the course of the therapy to make sure that you are not having any problems with the programs. If you give consent, we would like to record these phone calls so that we can supervise the member of staff making the calls. These tapes will be typed up and the transcripts read by members of the research team. All the information in these transcripts will be made anonymous. The tapes will then be destroyed.

If you do not consent to having these calls recorded, you can still participate in the trial.

After four months we will contact you again to ask you a further set of questions to see how you are feeling now. We will also do this at 12 months and 24 months after you have entered the study. We can interview you face-to-face or over the telephone to ask you these questions. Each of these follow-up visits or telephone calls will take about half an hour. Alternatively, we can send the questionnaires to you in the post with a stamped addressed envelope. You can then complete them and return them to us. You will be able to let us know how you would like to get the information to us.

We will need to collect some information from your medical records to find out what NHS services you have used while you have been participating in the study and if you have been prescribed any antidepressant medication. This will help us to work out which treatment is the best value for money. Overall the research study will last for four and a half years. Your involvement will only be for 24 months. At the end of the study we will send you a written summary of our findings, if you would like one.

Throughout the study you will continue to be looked after by your GP, as normal. You can see your GP as often as you and he/she thinks necessary

Expenses and payments

Unfortunately, we are not able to offer any expenses or payments to patients who participate in the study.

What will I have to do?

We would like you to be available for the interviews with our researchers, whether these are face-to-face or over the telephone. If you decide to receive follow-up questionnaires in the post, we would like you to complete these as fully as possible and return them to us. We would also like you to complete the treatment that you have been allocated to. You are able to take any prescribed or over-the-counter drugs. You are also free to try any other type of therapy for your depression.

What is the treatment that is being tested?

We are testing two different types of CBT packages, *Beating the Blues* and *MoodGYM,* against the usual treatment, GP care. Each of these involves a number of interactive therapy sessions and a small amount of 'homework' between sessions. We know from research already conducted that each of these are effective ways of treating depression, but we do not know which is the best or if they are all equally effective.

What are the alternatives for treatments?

People with depression are generally offered either antidepressant medication or some form of talking treatment (psychotherapy or counseling) delivered by a healthcare professional working in a GP practice. Sometimes people receive both medication and talking treatment. For various reasons, the availability of talking treatments is sometimes limited in the NHS, and patients sometimes have to wait until a healthcare professional is available to offer this treatment. By participating in this trial, you will still have access to all of these alternatives if the treatment that you are allocated does not work or is not one which you find helpful.

What are the possible disadvantages and risks of taking part?

We know from many research studies that usual GP care is generally effective in treating depression. The research supporting the use of computerised CBT has only been conducted more recently and we are less sure how effective computerised CBT is if added to usual GP care, and who it works best for. If you are allocated computerised CBT, we will ask you to try this treatment.

Computerised CBT has been designed to be easy to use, even if you have no experience of using computers. If you are allocated this treatment, you may not like this method of treatment. If this is the case, we may ask you more about this to help us understand people's experience of computerised CBT in more detail. You will still have access to the full range of treatment options that are available to other people in your GP practice throughout the trial if this is the case.

What are the side effects of any treatment received when taking part?

There are no known side effects of computerised CBT.

What are the possible benefits of taking part?

We cannot promise that the study will help you as we do not whether computerised CBT is better than the usual treatment.. However, we know from existing research that both computerised CBT and the usual treatment generally help people with depression. The information we get from this study will help improve the treatment of people with depression.

The computerised form of treatment has been officially recommended for use in the NHS, but we know that this form of treatment is still not universally available. By participating in this trial, you may well therefore get access to a treatment that might not otherwise be available in your GP practice or area.

What happens when the research study stops?

Our study is only funded for a limited period of time. When the study is completed, we will no longer be in a position to offer computerised therapy in your GP practice. If your GP practice has found the service helpful in improving access to care, then they may well wish to offer this treatment on a routine basis. We will have helped them to make it easier to set up a computerised CBT service during the time that they have participated in this study.

What if there is a problem?

We will address any complaint about the way you have been dealt with during the study or any possible harm you might suffer. Detailed information on this is given in Part 2.

Will my taking part in the study be kept confidential?

Yes. We will follow ethical and legal practice and all information about you will be handled in confidence. The details are included in Part 2.

This completes Part 1. If this information has interested you and you are considering whether to participate, please read the additional information in Part 2 before making any decision.

Part 2: Detailed Information

What if relevant new information becomes available?

Sometimes we get new information about the treatment being studied. If this happens, the research team will tell you and discuss whether you should continue in the study. If you decide not to carry on, your usual GP care will continue. If you decide to continue in the study, we may ask you to sign an

updated consent form. If we think you should withdraw from the study, we will explain the reasons and arrange for your care to continue.

If the study is stopped for any other reason, we will tell you and arrange your continuing care.

What will happen if I don't want to carry on with the study?

You are free to withdraw from the treatment that you are receiving at any time. If you do, it is still important for us to carry out the follow-up interviews with you. If you do not want to be interviewed, you are free to refuse. We would still like to be able to use the data that we have collected about you.

What if there is a problem?

If you have a concern about any aspect of this study, you should ask to speak to the researcher who will do their best to answer your questions (*contact number).* If you do not want to speak to the researcher you can contact the local principal investigator (*name and contact details)* or the chief investigator, Professor Simon Gilbody (telephone number: 01904 321370, email: simon.gilbody@york.ac.uk. If you remain unhappy and wish to complain formally, you can do this through the NHS Complaints Procedure. You can obtain details from your GP surgery.

In the event that something goes wrong and you are harmed during the research and this is due to someone's negligence then you may have grounds for a legal action for compensation against the University of York or your clinician.

Will my taking part in the study be kept confidential?

All information collected about you during the course of the research will be kept strictly confidential. We will keep the information we collect about you separate from your contact details and we will only link this information together with a secure code. We will store all information securely. At the end of the study, researchers from the University of York will need to have access to your medical records to see what NHS services you have used during the study.

We will use the information we collect to decide how effective each of the treatments are that we are testing. We will keep it for 20 years and then destroy it securely. We will destroy all contact information immediately after the end of the study.

You have the right to check the accuracy of data held about you and correct any errors.

Involvement of your GP

We will inform your GP if you agree to participate in the trial and if you decide to withdraw. We will also inform them about which treatment group you have been allocated to. He/she will need this information when looking after you. If, we are worried that you are having thoughts about harming yourself, we may need to discuss these with your GP. We will of course discuss this with you.

If you send us questionnaires through the post and we are worried that you are having thoughts about harming yourself, we will make every effort to contact you on the telephone to discuss these thoughts. If we are unable to contact you quickly, we will let your GP know of our concerns.

What will happen to the results of the research study?

We will publish the results of this research study widely. As well as producing a research report and writing articles for health professionals to read, you will be given a summary of the findings. We will ensure charities such as Depression Alliance are informed of the results of the trial. You will not be personally identified in any publications from this trial.

Who is organizing and funding the research?

The University of York is organizing this research. The funder is the Health Technology Assessment (HTA) Programme. We will pay your GP for the work that he/she and the practice staff do as part of the study.

Who has reviewed the study?

All research in the NHS is looked at by an independent group of people, called a Research Ethics Committee to protect your safety, rights, wellbeing and dignity. This study has been reviewed and given favourable opinion by Leeds (East) Research Ethics Committee.

Further information and contact details

For further general information about research, please see the INVOLVE website at http://www.invo.org.uk

For specific information about this research study, please contact

Liz Littlewood
Trial Manager
Area 4, Seebohm Rowntree Building
Department of Health Sciences
University of York
York, YO10 5DD

Telephone: 01904 321828
Email: liz.littlewood@york.ac.uk

For advice about whether you should participate, please speak to the local researcher or your own GP.

If you have a concern about any aspect of this study, please see the contact details given on Page 6 of this information sheet.

Thank you for reading this and for considering taking part in this study.
